# Epidemiologic potentials and correlational analysis of *Vibrio* species and virulence toxins from water sources in greater Bushenyi districts, Uganda

**DOI:** 10.1038/s41598-021-01375-3

**Published:** 2021-11-17

**Authors:** Hope Onohuean, Anthony I. Okoh, Uchechukwu U. Nwodo

**Affiliations:** 1 SA-MRC Microbial Water Quality Monitoring Centre, University of Fort Hare, Alice, 5700 South Africa; 2 Applied and Environmental Microbiology Research Group (AEMREG), Department of Biochemistry and Microbiology, University of Fort Hare, Private Bag 1314, Alice, 5700 Eastern Cape South Africa; 3 Biopharmaceutics Unit, Department of Pharmacology and Toxicology, School of Pharmacy, Kampala International University, Western-Campus, Ishaka-Bushenyi, Uganda

**Keywords:** Molecular biology, Environmental sciences

## Abstract

Adequate water supply is one of the public health issues among the population living in low-income settings. Vibriosis remain a significant health challenge drawing the attention of both healthcare planners and researchers in South West districts of Uganda. Intending to clamp down the disease cases in the safest water deprive locality, we investigated the virulent toxins as contaminants and epidemiologic potentials of *Vibrio* species recovered from surface waters in greater Bushenyi districts, Uganda. Surface water sources within 46 villages located in the study districts were obtained between June and October 2018. Standard microbiological and molecular methods were used to analyse samples. Our results showed that 981 presumptive isolates retrieved cell counts of 10–100 CFU/g, with, with (640) 65% confirmed as *Vibrio* genus using polymerase chain reaction, which is distributed as follows; *V. vulnificus* 46/640 (7.2%), *V. fluvialis* 30/594 (5.1), *V. parahaemolyticus* 21/564 (3.7), *V. cholera* 5/543 (0.9), *V. alginolyticus* 62/538 (11.5) and *V. mimicus* 20/476 (4.2). The virulence toxins observed were heat-stable enterotoxin (*stn*) 46 (82.10%), *V. vulnificus* virulence gene (*vcgCPI*) 40 (87.00%), extracellular haemolysin gene {*vfh* 21 (70.00)} and Heme utilization protein gene {*hupO* 5 (16.70)}. The cluster analysis depicts *hupO* (4.46% n = 112); *vfh* (18.75%, n = 112); *vcgCPI* and *stn* (35.71%, & 41.07%, n = 112). The principal component analysis revealed the toxins (*hupO, vfh*) were correlated with the isolate recovered from Bohole water (BW) source, while (*vcgCPI, stn*) toxins are correlated with natural raw water (NRW) and open springs (OS) water sources isolates. Such observation indicates that surface waters sources are highly contaminated with an odds ratio of 1.00, 95% CI (70.48–90.5), attributed risk of (aR = 64.29) and relative risk of (RR = 73.91). In addition, it also implies that the surface waters sources have > 1 risk of contamination with *vfh* and > six times of contamination with *hupO* (aR = 40, − 66). This is a call of utmost importance to the population, which depends on these water sources to undertake appropriate sanitation, personal hygienic practices and potential measures that ensure water quality.

## Introduction

The water niche is one of the essential nexuses of the ecosystem, which habitats numerous living organisms capable of causing diseases and spread pathogenic virulence toxins. Nevertheless, water forms the most extensive composition of the earth crust and utmost importance for every living thing.

*Vibrio* spp. is among the most naturally occurring bacteria in surface water sources that are of human concern^1,2^ since they are implicated in vibriosis infections. Vibriosis is a generalised term used to describe elevated *Vibrio* spp. and associated infections in the intestine. While cholera is toxin induce sickness caused by *Vibrio cholerae* exotoxins released into the intestine. However, communications have shown a substantial proportion of environmental strains to be harmless and exist as commensals of marine microbiota. Although some are labelled opportunistic pathogens in humans and aquaculture^3^, about 12 *Vibrio* species are recognised as pathogenic, causing human illness^1^. These human pathogenic *Vibrio* spp., produce an array of virulence genes or toxins linked to mild to fatal illnesses^4^. For instant, toxins including Cholera toxin, cytolysin *VvhA*, metalloprotease *Vvp*, flagella and *RtxA* toxin.

*Vibrio* Cholerae, the etiological agent of cholera, produces cholera toxin (*CTX*, *Ctx* or *CT*) and toxin-coregulated pilus (*TCP*) as the primary virulence determinants for pathogenicity. *CT* encodes the *ctxA* and *ctxB* genes located in the integrated prophage *CTX′* responsible for the severe loss of water and electrolyte^5^ diarrhoea infection. *TCP* encode for *tcpA*, utilised to colonies small intestinal epithelium by the bacterium^6^ regulated by the *toxR* regulon during expression in vivo^7^.

Skin infections and severe gastrointestinal disorders associated with *V. parahaemolyticus* pathogenicity are linked to the expression of thermostable direct hemolysin (*tdh*) and a gene associated with thermostable direct hemolysin (*trh*)^8,9^, as well the production of cytotoxic and enterotoxic effects^10^. *Vibrio Vulnificus* and *fluvialis* are referred to as emerging pathogens of humans. *Vulnificus* is encoded the virulence-correlated gene (*vcg*) implicated to cause wound infections, gastroenteritis or “primary septicemia”^11–13^. However, the degree of virulence of *V. vulnificus* is related to the origin of the strain; thus, clinical strains are more virulent than environmental isolates^14^.

*Vibrio fluvialis* produce several compelling toxins, such as the stable heat enterotoxin (*stn*)^15^, cell elongation factor or components like Chinese hamster ovary (CHO) or CHO cell-killing factor, lipase, cytotoxin, hemolysin and protease^16–18^ although their roles in pathogenesis are not well established. *Vibrio fluvialis* virlence manifest as hemorrhagic cellulites and cerebritis^19^, peritonitis^20^, acute otitis^21^, biliary tract infection^22^, bacteraemia^23^ and ocular infections^24^. Other virulence factors associated with *fluvialis* include but are not limited to the *V. fluvialis* protease gene (*vfp*), heme utilisation protein gene (*hupO*), extracellular haemolysin gene (*vfh*), and heme utilisation protein gene (*hupO*)^17^.

The virulence *toxR* in cholerae and *fluvialis* is specifically implicated in the bile resistance and the initial phase of vibriosis disease establishment. Among the human pathogens include halophilic *Vibrios*, *V. alginolyticus*, and *V.* *metschnikovii* as well as *Vibrio* *mimicus* via the production of virulence. Other species such as *Vibrio* alginolyticus have been reported as probiotics for shrimp aquaculture^25,26^. *Vibrio* parahaemolyticus Shrimp-associated gastroenteritis has been reported^27^, and some of the pathogenic *Vibrio* species were associated with shrimp infections^28^. The manifestation of virulence determinants is key to distinguishing between the probable non-virulent strains from potentially virulent *Vibrio* strains of clinical importance. Generally, the clinical significance of *Vibrio* spp. is associated with drinking contaminated water or consuming raw or improperly cooked seafood^29^.

The cholera toxin A subunit^30^ and the El Tor cytolysin/haemolysin is activated by the proteolytic effect metalloprotease of *Vibrio* cholerae, commonly called haemagglutinin/protease (*Hap*) seen in cholera pathogenesis. The cascade event leads to hydrolysing of important physiological proteins, enhancing dissociation, mucin gel penetration and consistent infection spreading through the gastrointestinal tract. *VFH* forms pores in the erythrocyte membrane, which is more significant than those generated by other Vibrio hemolysins such as *Vibrio* cholera, *Vibrio* parahaemolyticus, and *Vibrio Vulnificus*^7^ implicated in the bloody occurred diarrhoea in some patients. Therefore, *vfp* could be a pathogenic factor in *V.* *fluvialis* because of its similar biological activity to metalloproteases seen in *V.* *cholerae* and *V.* *vulnificus*.

The amount of reported *Vibrio* species and their associated toxins has increased rapidly in the last decades^11,31–33^. However, in Uganda, only outbreak cases of *Vibrio cholera* is reported with no information on other species and a dearth of studies on *Vibrio* toxin recovered from the surface water uses.

Though the percentage of the tap water available for use by different communities has increased, that of the districts of greater Bushenyi is still far below the percentage that will meet the people's minimum needs. The social, economic, and cultural bond between the local communities and the available ponds, lakes, springs and streams continue to increase strength. It becomes challenging to isolate the people and these waters for any significant organised study, especially in times of epidemic. There may be epileptic reports and studies at the national level about *Vibrio* associated diseases but grass root coordinated surveillance to inform policy updates that can lead to effective control of this disease is grossly inadequate and, in some cases, lacking. There is a sustained significant upsurge in the reports of *Vibrio* associated and diarrheagenic disease conditions both in the districts of greater Bushenyi and the country at large. This indicates that the national and local control mechanisms may be wanting in their expected capacity to clamp down these diseases conditions that have continued to ravage the ordinary citizens in remote hard to reach areas where the significant population still fall sick, get worst and die without access to medical services and interventions^34,35^.

Nevertheless, new pathogenic strains and virulence continue to emerge in endemic, pandemic and spreading to another region. It is worrisome that the precise role of most *Vibrio* spp., pathogenicity determinants in producing the clinical manifestations remains unclear.

These clearly calls for coordinated local and national response to make society a better place to be. Based on these premises, this study was designed to investigate the epidemiologic potentials of *Vibrio* species virulence toxins recovered from surface waters in greater Bushenyi districts, Uganda. The correlations association between the level of virulence toxins contamination and water sources were analysed.

## Materials and methods

### Study locations

**Figure 1:**
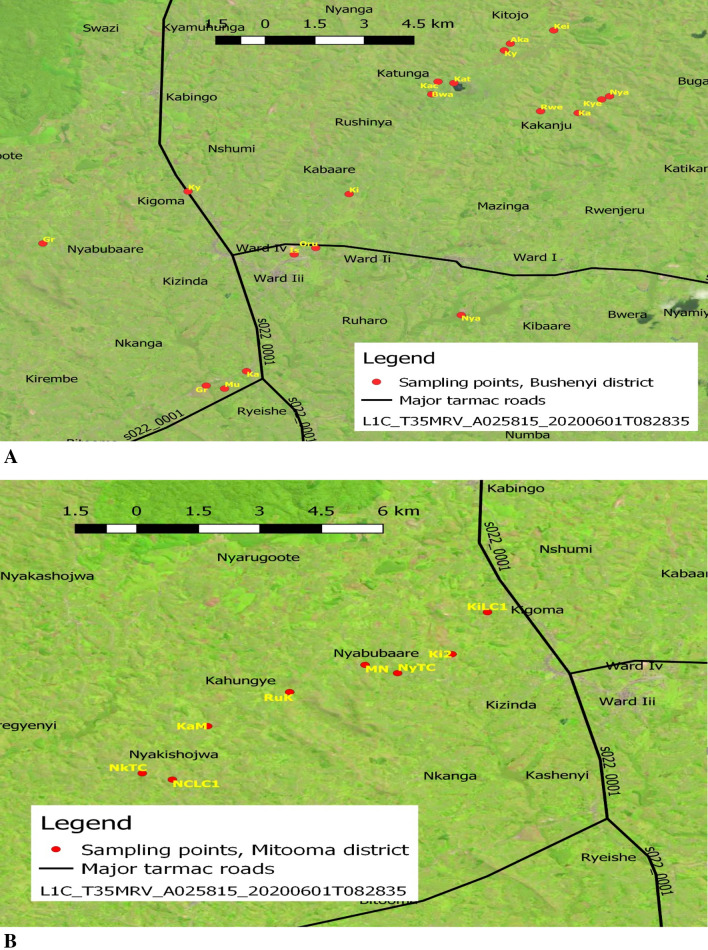

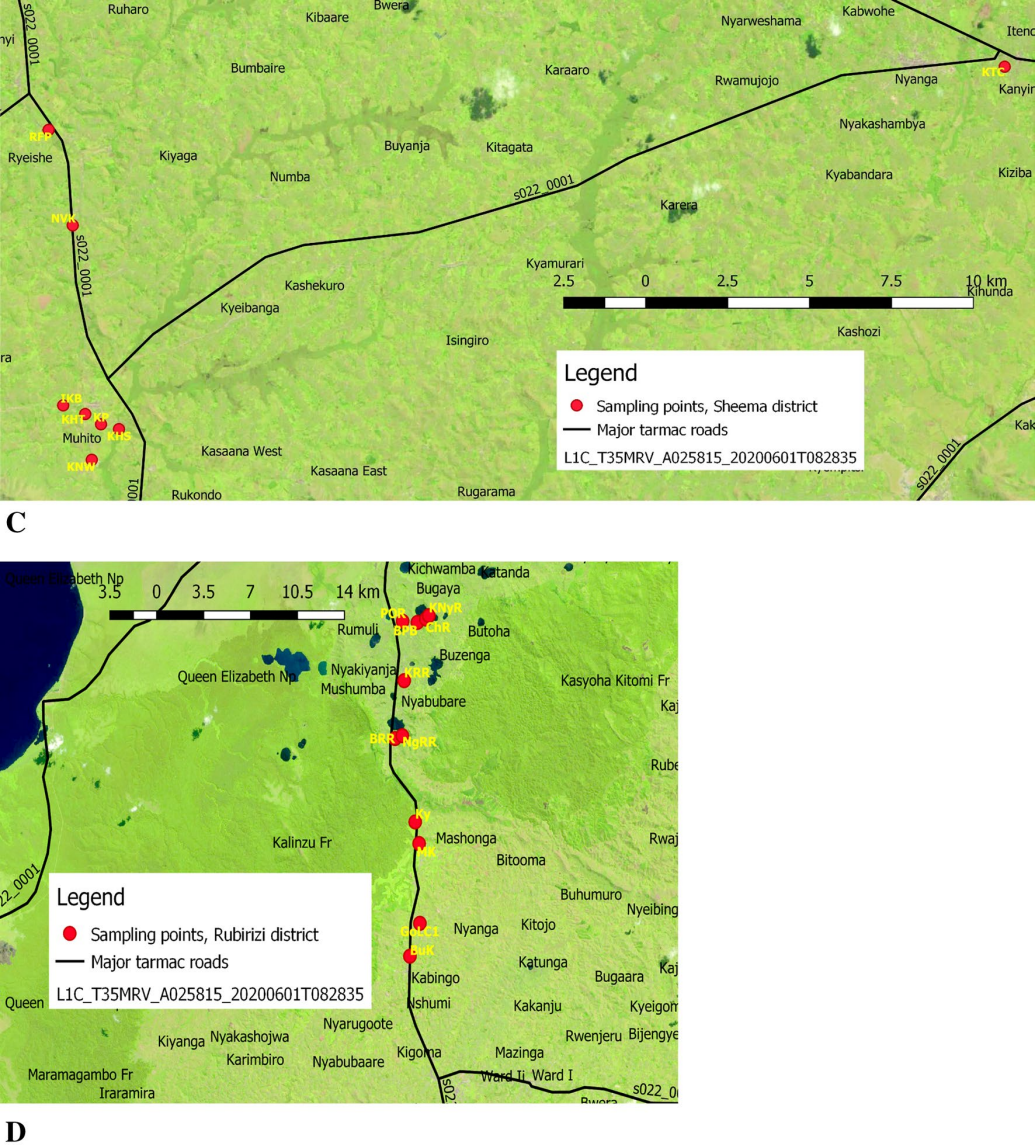
(**A**) Sampling locations (Bushenyi district). Ishaka; Is, Kashenyi spring 1; Ka, Ground water Kashenyi bridge; Gr, Mutanoga LC1 spring Mu, kyasima spring Bushenyi kigoma; Ky, Nyaruzinga Bushenyi natural raw water; Nya, Ground water; Gr, Orushenyi Ishaka spring; Oru, Kijumo kakaju igara Bushenyi; Ki, Bwangeme B Kakanju Bushenyi; Bwa, kacence Katungu kakajuigara spring gara west Bushenyi; Kac, Katungn Lake Bushenyi; Kat, kyantangu kakajuigara igara west Bushenyi; Ky, Akainje Igara spring West Bushenyi; Aka, keijengye Kakanju Igara East Bushenyi; Kei, Nyamwerande Igara East Bushenyi; Nya, kyemengo spring Igara East Bushenyi; Kye, kagogo nyamwerande Bushenyi; Ka, Rwengoma Kakanju igaraweast Bushenyi well water; Rwe. (**B**) Sampling locations (Mitooma district). Kirera village well LC1; KiLC1, Kirera 2; Ki2, Nyabubare town council tap; NyTC, Matimba Nyabubare village spring; MN, Rutundu village kahungye; RuK, Karoza Mitooma; KaM, Nkinga town council well water Mitooma; NkTC, Nyakishogwa central LC1 spring; NCLC1. (**C**) Sampling locations (Sheema district). Kabwohe_Itendero Town council; KTC, Rendez fish pond Kitagata; RFP, Nyakatooma village Kibingo; NVK, Ibanga Kitagata Bundumu; IKB, Kitagata hospital tap water; KHT, Kitagata natural raw water; KNW, Kitagata pond; KP, Kitagata Hot spring; KHS. (**D**) Sampling locations (Rubirizi district). Butare kyamunhuga well water; BuK, Gongo LC1; GoLC1, Mukayembe Kyamuhunga tap; MK, Ground water kyamuhunga; Ky, Bunyaruguru tap county Rototo Rubirizi; BRR, Lake Ngunguta Rototo Nyarugru Rubirizi; NgRR, Kasoga Rototo spring Rubirizi; KRR, Buhera Parliament village Buyaguru tap Rubirizi; BPB, Lake Chema Rubirizi; ChR, Lake/Cave kamwero Nyarugoro Rubirizi; KNyR, Rubirizi post office tab; POR.

The surface waters sources used in the four districts of the Western region of Uganda, including Bushenyi, Mitooma, Rubirizi and Sheema, were sampled for epidemiologic potentials of six pathogenic *Vibrio* species and virulence toxins. According to WHO, standard classification for drinking-water quality^36^. The surface waters used such as tap water, groundwater (borehole, open spring, ground running water, raw water, well water), Lakes, and fish pond were sampled from each of the 19 points in Bushenyi, 8 points in Mitooma, 11 points in Riburizi, and 8 points in Sheema districts as shown in Fig. 1A–D. Focus group discussion^37^ was organised with stockholders that assisted in getting to the study's sampling identification. The group comprising (investigators, interpreters, microbiologists, district health officers, village local chairperson (LC1s)) were selected base on their relevance to this study. To be more specific, the investigators outlined the purpose and objectives of the study; the district health officer assisted in suggesting the sampling points that fit the purpose of the study. The microbiologist provided the advice that ensured that sampling was aseptic. The LC1s confirmed that the water for sampling is present in the 46 villages and nominated the village health teams (VHTs). After the focus group discussion, the VHTs nominated by the LC1s took the investigators to the sampling points as shown in Fig. 1A–D.

The maps of the study areas in (Bushenyi; Mitooma; Rubirizi; and Sheema districts) was created using the open-source software QGIS desktop version 3.0.3^38^. The United States Geographical Surveys (USGS) provided the Sentinel-2 image ID: L1C T35MRV A025815 20200601T082835 dated 2020/9/7, which was overlay on a shapefile for Uganda and highways, +e satellite image file was adjusted to show land vegetations.

### Collection of samples, processing and enumeration of *Vibrio* spp.

A total of 46 villages was visited to obtain samples between June 2018 and October 2018.

Using sterilised Nalgene glass bottles (1000 ml) water samples were salvaged for four months in different sampling points each of the four Districts (Bushenyi, Mitooma, Rubirizi and Sheema) of South West of Uganda and conveyed on an ice-cool box to the department of medical microbiology laboratory, Kampala International University, Western-campus, Uganda for analysis within 6 h. Ten-folded dilution was carried out on the water samples as described by^39^, three series (10–1, 10–2 and 10–3) of which 1 ml of each dilution was spread plated onto thiosulphate citrate bile salts sucrose (TCBS) agar (Neogen, Lansing, MI 48912 USA) in triplicates for 24 h at 37 °C. The presumptive *Vibrio* spp., counted and expressed in colony-forming units per millilitres (CFU/ml) of water samples for the yellow and green colonies identified by colonial morphology and cultural characteristic of the colony^40,41^. A single colony of the presumptive Isolates was subsequently subcultured onto nutrient agar to ascertain purity; each sample’s pure culture was picked and stored in glycerol stock for further analysis at the Applied and Environmental Microbiology Research Group (AEMREG) laboratory, Department of Biochemistry and Microbiology, University of Fort Hare, South Africa.

### Molecular confirmation of presumptive *Vibrio* genus and delineation into six pathogenic *Vibrio* species

The glycerol stocks were resuscitated using nutrient broth (Merck, Modderfontein, South Africa) and incubation for 24 h at 37 °C, while the genomic DNA of the 981 presumptive *Vibrio* spp., isolates were extracted following the boiling procedure described by Refs.^42,43^ with modifications. The fresh overnight cultured isolates were subcultured into a sterile 1.5 ml microfuge tube and centrifuged (HERMLE, Siemensstr-25, D-78564 Wehingen, Germany) at a speed of 13,000 rpm for 10 min. The cells pellet was washed twice with phosphate-buffered saline, suspended on 500 µl sterile distilled water, and then lysed to release the DNA by boiling at 100 °C for 10 min pre-heated heating block (Techne heating block Dri-Block, DB-3D; Gauteng, Pretoria, South Africa). Afterwards, the suspension was centrifuged for 5 min at 15,000 rpm, and the supernatant was carefully pipetted into sterile Cryon tubes and stored at − 20 °C pending for use as a genomic DNA template PCR assays.

The primer pair F-5′CGG TGA AAT GCG TAG AGA T-3′ and R-5′TTA CTA GCG ATT CCG AGT TC-3′ previously described by Ref.^3^, was purchased from Inqaba Biotechnical Industries (Pty) Ltd., Pretoria, South Africa and used to amplify 16s RNA genes of *Vibrio* spp. with the amplicon size of 663 which was further delineated into the six pathogenic *Vibrio* species using the primers and condition in Table 1. The PCR reaction mixture of 25 µl (12 µl PCR master mix (New England BIOLABS), 1 µl of each forward and reverse primers, 6 µl of PCR grade water and 5 µl of genomic DNA template were amplified using BioRad T100 thermal Cycler Lasec. (621BR44012, Singapore). Afterwards, 4 µl of the amplicons were electrophoresed in 1.5% agarose gel using the thermal tank (Labnet, Enduro Gel XL, USA) on staining with ethidium bromide (0.5 µl) and 0.5× Tris–borate EDTA (TBE) buffer with a controlled base size of 100-bp DNA ladder (New England BIOLABS), Madison, WI, USA). A 100 Volt and 60 min electrophoresis process was done, and the gels were visualised under the UV trans-illuminator (Alliance 4.7, UVItec), Merton, London, UK.

**Table 1:** PCR condition and primer sets used for the screening of *Vibrio* spp.

Species	Primer	PCR primer sequence (5′–3′)	Amplicon size (bp)	PCR cycling condition	References
*Vibrio*	*16S rRNA*	CGGTGAAATGCGTAGAGAT TTACTAGCGATTCCGAGTTC	663	Initial denaturation (94 °C for 5 min), denaturation (94 °C for 30 s), 35 cycles, annealing (52 °C for 30 s), extension (72 °C for 60 s), final extension (72 °C for 10 min)	^44^
*V. parahaemolyticus*	*toxR*	TGTACTGTTGAACGCCTAA CACGTTCTCATACGAGTG	503	Initial denaturation (94 °C for 5 min), denaturation (94 °C for 30 s), 35 cycles, annealing (55 °C for 30 s), extension (72 °C for 60 s), final extension (72 °C for 10 min)	^45^
*V. vulnificus*	*vvhA*	ACTCAACTATCGTGCACG ACACTGTTCGACTGTGAG	366	Initial denaturation (94 °C for 5 min), denaturation (94 °C for 30 s), 35 cycles, annealing (55 °C for 30 s), extension (72 °C for 30 s), final extension (72 °C for 10 min)	
*V. cholerae*	*toxR*	GAAGCTGCTCATGACATC AAGATCAGGGTGGTTATTC	275	Initial denaturation (94 °C for 10 min), denaturation (94 °C for 60 s), 35 cycles, annealing (59 °C for 60 s), extension (72 °C for 1 min), final extension (72 °C for 10 min)	^46^
	*OmpW*	CACCAAGAAGGTGACTTTATTGTG GGTTTGTCGAATTAGCTTCACC	304		
*V. fluvialis*	*toxR*	GGATACGGCACTTGAGTAAGACTC GACCAGGGCTTTGAGGTGGACGAC	217	Initial denaturation (94 °C for 5 min), denaturation (94 °C for 60 s), 35 cycles, annealing (57 °C for 60 s), extension (72 °C for 90 s), final extension (72 °C for 7 min)	^47^
*V. alginolyticus*	*Vg gyrB F* *Vg gyrB R*	GAGAACCCGACAGAAGCGAAG CCTAGTGCGGTGATCAGTGTTG	338	Initial denaturation (93 °C for 5 min), denaturation (92 °C for 30 s), 30 cycles, annealing (56 °C for 1 min), extension (72 °C for 1.5 min), final extension (72 °C for 7 min)	^48^
*V. mimicus*	*VM-F* *VM-R*	CAGGTTTGCTGCACGGCGAAGA CCTTGAAGAAGCGGTTCGTGCA	177	Initial denaturation (93 °C for 5 min), denaturation (92 °C for 30 s), 30 cycles, annealing (57 °C for 1 min), extension (72 °C for 1.5 min), final extension (72 °C for 7 min)	

### Evaluation of virulence genes signature of *Vibrio* spp. recovered isolates

The virulence genes signature distribution in the confirmed *Vibrio* spp. isolates using PCR technique as described by^49,50^ with modifications. The sets of primers indicating the targeted genes, sequence and conditions are presented in Table 2. The genomic DNA templates of confirmed isolates of *Vibrio* spp., including (62-*Vibrio* alginolyticus, 30-*Vibrio fl*uvialis, 46-*Vibrio vulnificus*, 20-*Vibrio mimicus,* 21-*Vibrio parahaemolyticus,* 5-*Vibrio cholerae*) recovered from surface waters by adopting the earlier reported protocol described by Refs.^43,51,52^ and the PCR reaction mixture was made up to a final volume of 25 μl while the amplified amplicons were electrophoresed and visualised as stated earlier.

**Table 2:** PCR condition and primer sets used for the screening of *Vibrio* virulence toxins (genes).

Virulence toxin (genes)	PCR primer sequence (5′–3′)	Amplicon size (bp)	PCR cycling condition	References
*tdhF*	GGTCTAAATGGCTGACATC	199	Initial denaturation (93 °C for 5 min), denaturation (92 °C for 30 s), 35 cycles, annealing (55 °C for 60 s), extension (72 °C for 60 s), final extension (72 °C for 7 min)	^53^
*tdhR*	CCACTACCACTCTCATATGC			
*trhF*	CATTTCCGCTCTCATATGC	250		
*trhR*	GGCTCAAAATGGTTAAGCG			
*vcgCP1*	AGCTGCCGATAGCGATCT	278	Initial denaturation (93 °C for 5 min), denaturation (94 °C for 40 s), 35 cycles, annealing (56 °C for 40 s), extension (72 °C for 60 s), final extension (72 °C for 7 min)	^54^
*vcgP3*	CGCTTAGGATGATCGGTG			
*vcgEP2*	CTCAATTGACAATGATCT	278	Initial denaturation (94 °C for 5 min), denaturation (94 °C for 40 s), 35 cycles, annealing (49 °C for 40 s), extension (72 °C for 60 s), final extension (72 °C for 7 min)	
*vcgP3*	CGCTTAGGATGATCGGTG			
*vfh-F*	GCGCGTCAGTGGTGGTGAAG	800	Initial denaturation (94 °C for 15 min), denaturation (94 °C for 40 s), 35 cycles, annealing (50–60 °C for 40 s), extension (72 °C for 60 s), final extension (72 °C for 7 min)	^17^
*vfh-R*	TCGGTCGAACCGCTCTCGCTT			
*hupO-F*	ATTACGCACAACGAGTCGAAC	600	Initial denaturation (93 °C for 15 min), denaturation (92 °C for 40 s) 35 cycles, annealing (50–62 °C for 60 s) extension (72 °C for 90 s) final extension (72 °C for 7 min)	^17^
*hupO-R*	ATTGAGATGGT AAACAGCGCC			
*vfpA-F*	TACAACGTCAAGTTAAAGGC	1790		
*vfpA-R*	GTAGGCGCTGTAGCCTTTCA			
*stn-F*	GGTGCAACATAATAAACAGTCAACAA	375		
*stn-R*	TAGTGGTATGCGTTGCCAGC			

### Safety for research staff and environment

After the entire experiment, all specimens and isolates were decontaminated using autoclave at 121 °C, 15 PSI for 15 min. The decontaminated specimen and isolate were incinerated, and the ash was buried at the designated spot.

### Statistical analysis

The result was entered into Microsoft excel. The distribution of toxins in the water sources was analysed using the violin box plot by considering Dunn's post hoct test to compare the occurrence of virulence toxin across the surface waters sources. Using multi-cluster analysis and Spearman's correlation coupled with Principal Component Analysis (PCA) was used to understand the correlations between the *Vibrio* spp., virulence toxins and surface water sources. All in RStudio version 3.5.1 software^55^. Furthermore, the significant epidemiological prevalence and risk estimate of toxins contaminations at 95% confidence interval evaluated in WINPEPI software version 11.65^56^. All statistical significant differences were recognised at p < 0.05.

### Ethical consideration

The protocol for this study was reviewed by the research ethics committee of the Kampala International University, Western-Campus, Uganda, and obtained a clearance number of Nr.UG-REC-023/201919.

## Results

### The Surface waters sources studied in the region

South-Western Uganda is endowed with water resources and forest game reserved. The temperature ranges relatively between 19 and 24 °C through the year, even with the seasonal variations. We identified and studied nine primary types of surface waters sources used by the populaces in 46 villages of the greater Bushenyi districts. These surface waters were surveyed for the distribution and prevalence of *Vibrio* spp. and virulence toxins. The surface waters used by the general population across numerous purposes of life from domestic to agricultural uses and recreational/medical tourist attractions in the districts are highlighted in Table 3. The map of sample collection points in the four districts are shown in Fig. 1A–D.

**Table 3:** Surface waters sources sampled in the districts.

Surface waters sources	Uses
Bohole water	Irrigation and domestic use
Fish pond	Fishing and farming
Ground running water	Car washing, farming, animal rearing and domestic use
Hot spring	Domestic use, spiritual and recreational purposes
Lake	Fishing, farming, irrigation, swimming, animal rearing, and domestic use
Natural raw water	Animal rearing, irrigation, national treatment and supply for domestic use
Open springs	Domestic uses, irrigation and farming
Tap water	Domestic uses, irrigation and farming
Well water	Domestic uses and farming

Author compilation as found in the districts.

### The total *Vibrio* cell densities count from the surface waters used in the districts

**Figure 2:**
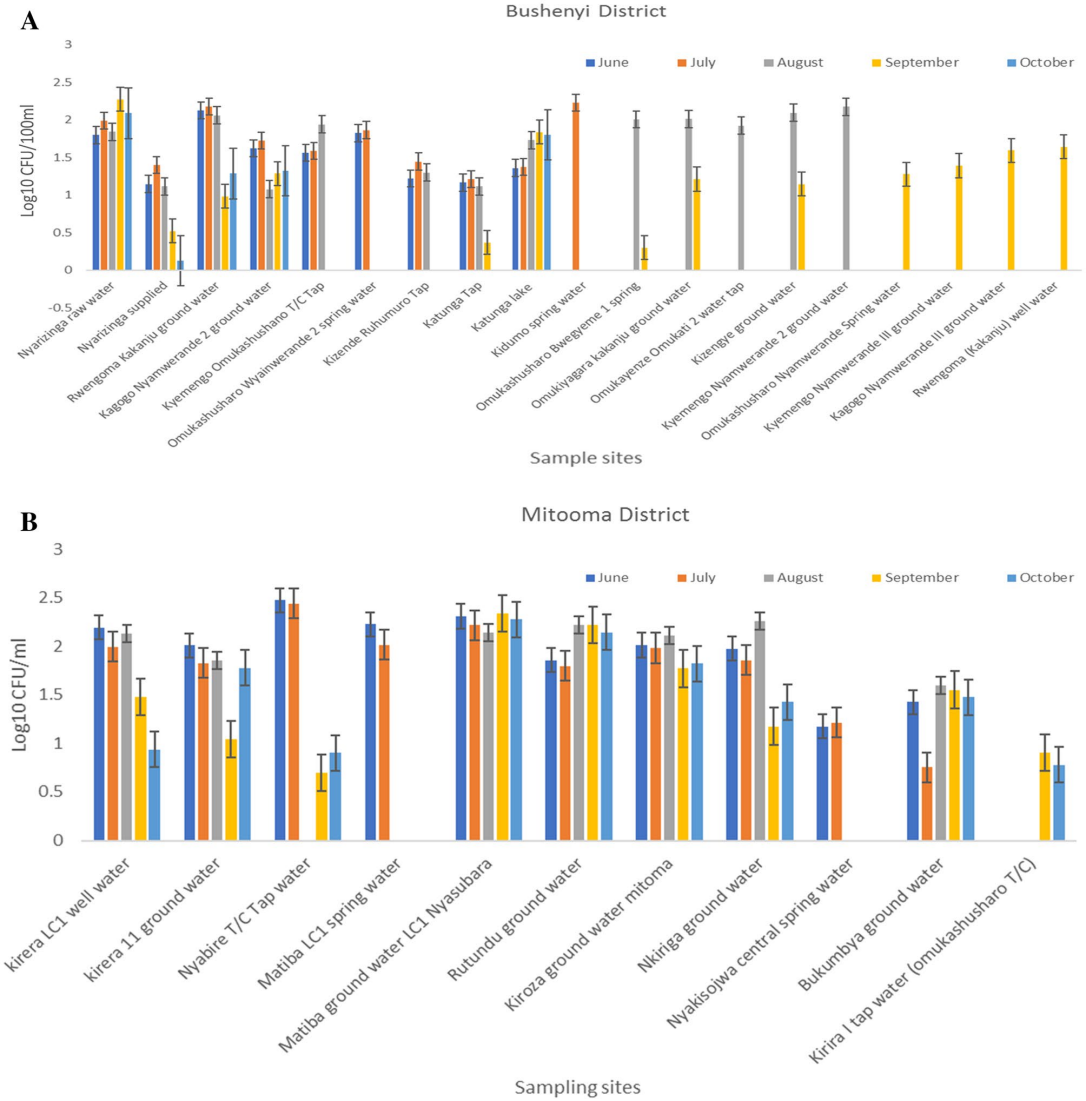

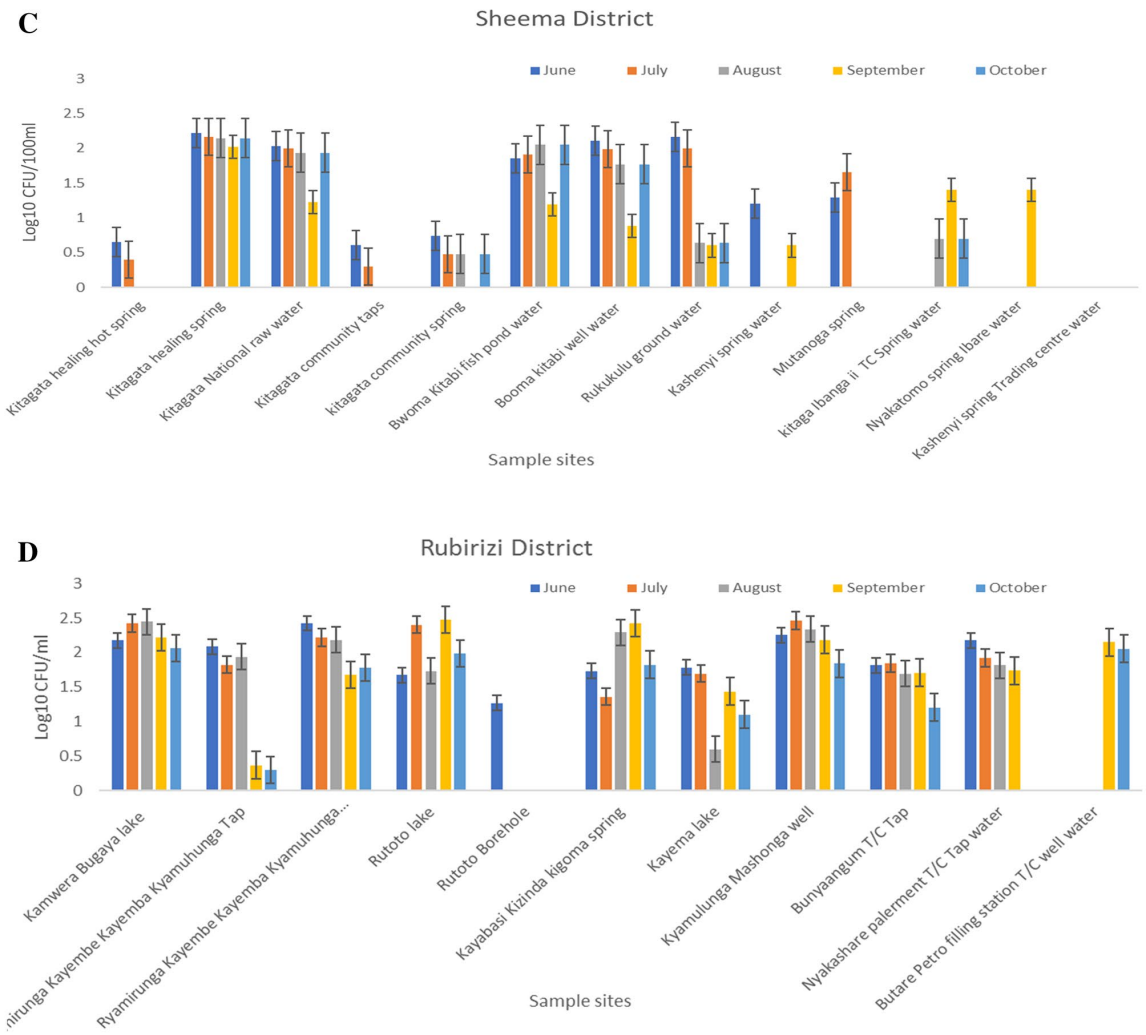
(**A**) Presumptive *Vibrio* counts from 19 selected sampling points in Bushenyi subcounties. The graph shows a plot of the seasonal average of log10 CFU/100 ml for *Vibrio* counts over the period of June 2019–October 2019. (**B**) Presumptive *Vibrio* counts from 11 selected sampling points in counties of Mitooma Districts. The graph shows a plot of the seasonal average of log10 CFU/100 ml for *Vibrio* counts over the period of June 2019–October 2019. (**C**) Presumptive *Vibrio* counts from 13 selected sampling points in counties of Sheema Districts. The graph shows a plot of the seasonal average of log10 CFU/100 ml for *Vibrio* counts over the period of June 2019–October 2019. (**D**) Presumptive *Vibrio* counts from 11 selected sampling points in counties of Rubirizi Districts. The graph shows a plot of the seasonal average of log10 CFU/100 ml for *Vibrio* counts over the period of June 2019–October 2019.

The mean of presumptive *Vibrio* cell densities counts from the surface Waters from the districts are expressed in log10 CFU per gram. The results showed ranged values of 0.125–2.231 log10 in Bushenyi district; the sampling points have no even *Vibrio* cell counts. There are almost cell counts in all the sampling points in August and September except for few points. In the Mitooma district, the months has relatively moderate cell counts across the sampling points, with some months without cell counts in some sampling points. However, the cells count range from 0.753 to 2.474 log10. Also, in the Sheema district, the *Vibrio* cell counts range from 0.397 to 2.215 log10. There were relatively cell counts in all the sampling, only the month of September has cells count in the sampling point Nyakatomo Ibare open spring and no cell count in Kashebyi trading centre open spring. In the Rubirizi district, the cells count range from 0.301 to 2.426 log10. Rutoto borehole only has cells count in June, while the Butare town council well only has cells count for September and October. This implies that presumptive *Vibrio* species are high in some water in other months, as we see in the mean cell count in Fig. 2A–D.

### The distribution of six pathogenic *Vibrio* spp. in surface waters in greater Bushenyi districts

**Figure 3:**
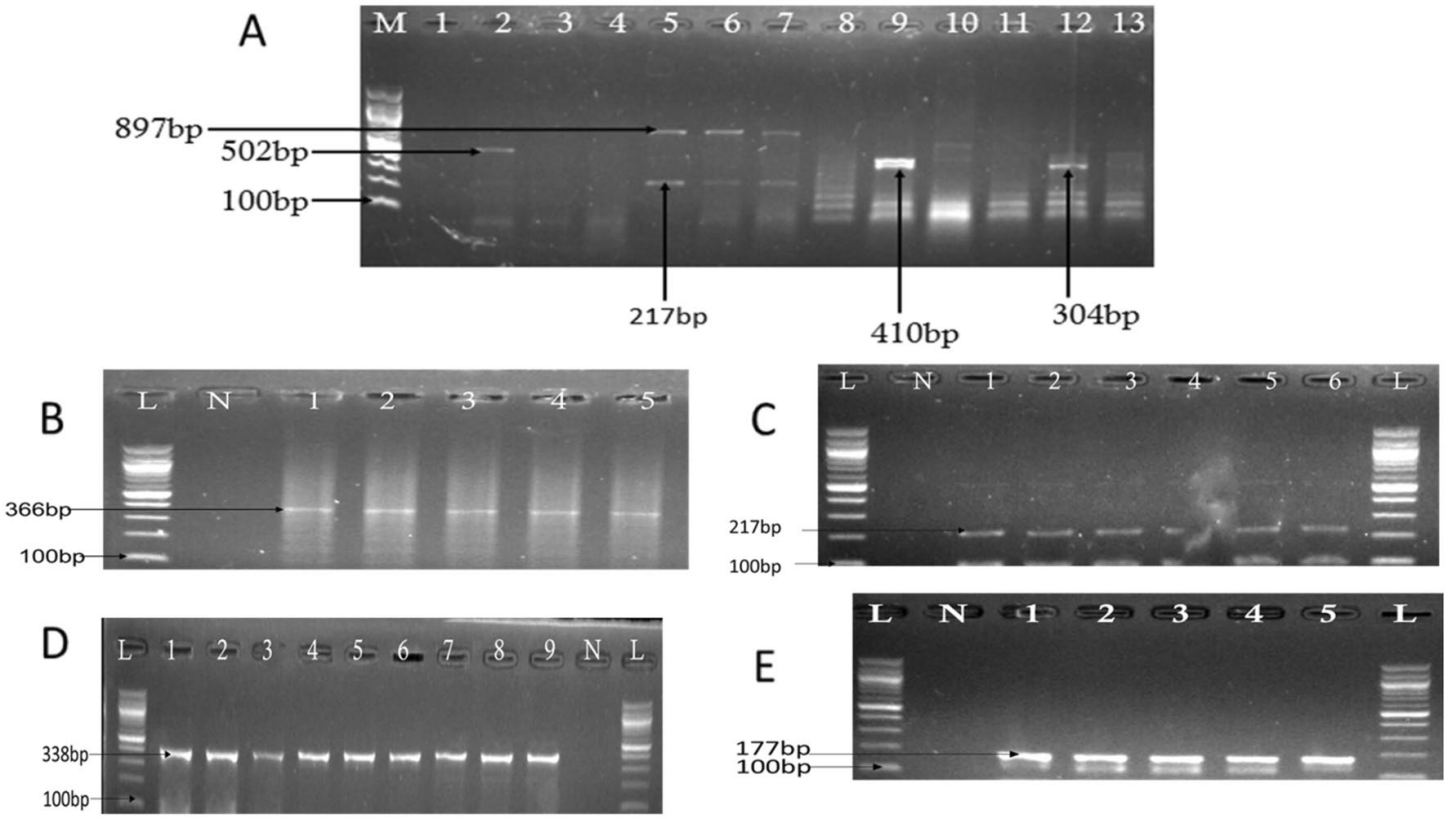
(**A**–**E**) Gel picture representing molecular confirmation of the pathogenic *Vibrio* species. For (**A**), Lane M: Molecular Marker (100 bp); Lane 1: Negative control; Lane 5, 6, 7 (*V. parahaemolyticus (toxR*) and *V. fluvialis* (*toxR*)); Lane 12, 13: *V. cholerae* (*ompW*). While (**B**–**E**), Lane L: Molecular Marker (100 bp); Lane N: Negative control and 1, positive control. (**B**) = *vulnificus* (*vvhA*), (**C**) = *fluvialis* (*toxR*), (**D**) = *alginolyticus* (*Vg gyrB*), (**E**) = *mimicus* (*VM*).

Out of 981 presumptive isolates, (640) 65% were confirmed to be *Vibrio* genus using the polymerase chain reaction analysis as we reported^57^ and gel picture in Supplementary S1. The result of the distribution of the six pathogenic *Vibrio* species recovered from the water sources includes; *V. vulnificus* 46/640 (7.2%), *V. fluvialis* 30/594 (5.1%), *V. parahaemolyticus* 21/564 (3.7%), *V. cholerae* 5/543 (0.9%), *V. alginolyticus* 62/538 (11.5%), *V. mimicus* 20/476 (4.2%) Table 4 and gel pictures in Fig. 3A–E.

**Table 4:** Distribution of six human pathogenic *Vibrio* spp. by PCR, Risk estimate (relative risk and attributable risk), odds ratios in surface waters of greater Bushenyi Districts.

Human pathogenic* V. species*	Pathogens	95% CI (LL–UL) prevalence	RR	aR	Odds ratios	p-value
*V. vulnificus*	46 (7.2)	5.37–9.39	0.08	− 85.63	1	–
*V. fluvialis*	30 (5.1)	3.5–7.04	0.05	− 89.9	0.687	0.463
*V. parahaemolyticus*	21 (3.7)	2.38–5.54	0.04	− 92.55	0.499	0.039
*V. cholerae*	5 (0.9)	0.34–2.03	0.01	− 98.16	0.12	0
*V. alginolyticus*	62 (11.5)	9.03–14.43	0.13	− 76.95	1.682	0.051
*V. mimicus*	20 (4.2)	2.66–6.30	0.04	− 91.6	0.566	0.156

*CI* confidence interval, *LL* lower limit, *UL* upper limit, *RR* relative risk, *aR* attributed risk.

### Molecular identification and distribution of virulence toxins in the surface waters sources used in the region

**Figure 4:**
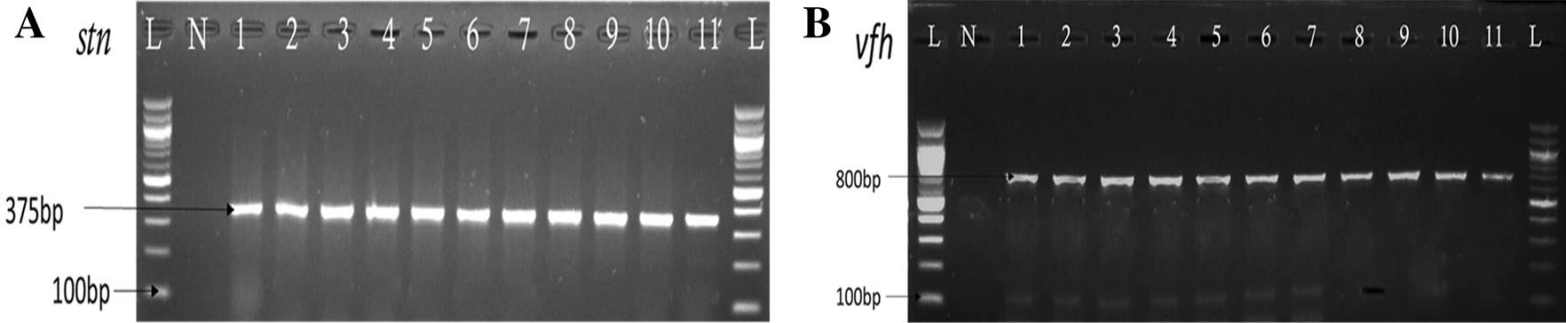
Gel picture representing molecular characterisation of virulence toxins. (**A**) (*stn* virulence toxins) Lane 1–11 positive, (**B**) (*vfh* virulence toxins) Lane 1–11 positive. Lane L: Molecular Marker (100 bp); Lane N: Negative control.

**Figure 5:**
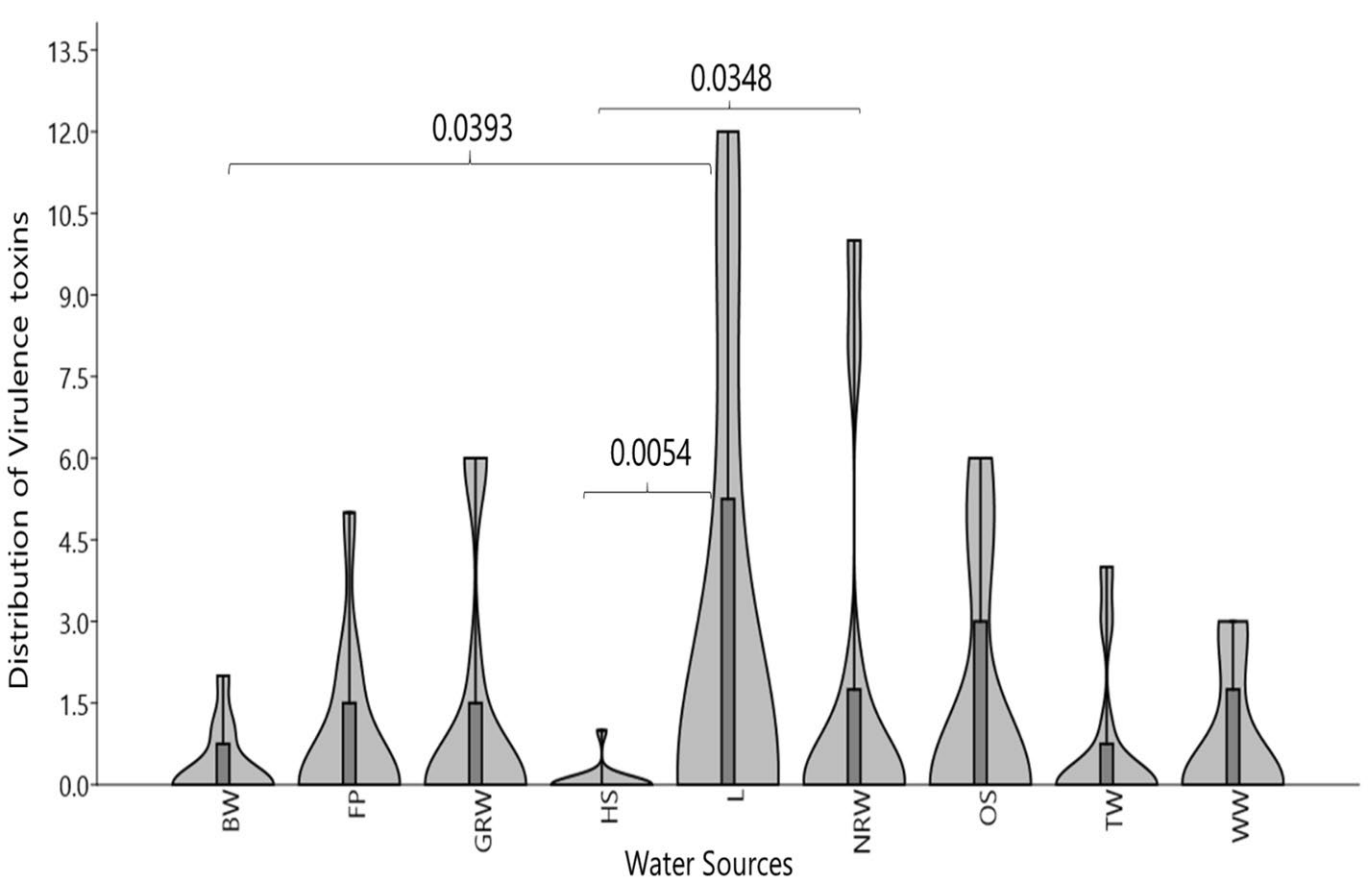
Distribution of *Vibrio* spp. Virulence determinants varied significantly on the surface waters in the districts by (Dunn's post hoct, p ≤ 0.05). *BW* Bohole Water, *FP* fish pond, *GRW* ground running water, *HS* Hot Spring, *L* Lake, *NRW* natural raw water, *OS* open Springs, *TW* tap water, *WW* well water.

Of the 316 *Vibrio* spp. strains screen for virulence toxins, 112 (35.44%) were positive to molecular PCR techniques. The gel pictures of the molecular characterisation of the majority of the virulence toxin are shown in Fig. 4. The results of the distributions of the toxins in the study surface waters reveal that Lake surface water sources harbors virulence toxins 31 (27.68%) and natural raw water sources 21 (18.75%) and open springs water sources 15 (13.39%) with the Hot Spring least of 1 (0.89). The occurrence of virulence toxins in the Surface waters sources varied significantly in distribution (BW and L, Dunn's post hoct, p = 0.0393, HS and L, p = 0.0054, HS and NRW, p = 0.0348). The amalgamated violin and box plots, violin expanse, displays the distribution by adding the mild and extreme outliers. The box plot displays the median at concentration ellipse of 25–75% percentiles in Fig. 5.

### Prevalence and epidemiological significance of virulence toxins in the surface waters sources used in the region

The pathogenicity of Vibrioses is tied to the expression of the virulence toxins leading to the unending endemic infections in population and region. Among the twelve pathogenic endemic virulence toxins, heat-stable enterotoxin (*stn*) 46 (82.10%), *V. vulnificus* virulence genes (*vcgCPI*) 40 (87.00%) were found to be prevalent in surface waters sources, showing that the surface waters sources are highly contaminated with an odds ratio of 1.00, 95% CI (70.48–90.5), attributed risk of (aR = 64.29) and relative risk of (RR = 73.91). In addition, the Extracellular haemolysin gene *vfh* and Heme utilisation protein gene *hupO* prevalence was 21 (70.00) and 5 (16.70) implies that the surface waters sources has > 1 risk of contamination with *vfh* and > six times of contamination with *hupO* (aR = 40, − 66). Nevertheless, all other virulence toxins investigated in this study showed a 0.00% prevalence and no contamination (aR = − 100). The burden of risk of *vcgCPI* virulence toxin contaminations of the surface waters sources is of significant concern to the public (RR = 6.67) identified as shown in Table 5.

**Table 5:** Prevalence distribution of virulence toxins of human pathogenic *Vibrio* spp. by PCR (relative risk and attributable risk), odds ratios in surface waters of each district.

Name of virulence toxins	Genes	Isolate tested	Virulence toxins	95% CI of p LL; UL	RR	aR	Odds ratios	p-value
Heat stable enterotoxin	*stn*	56	46 (82.10)	70.48–90.56	4.60	64.29	1.00	–
Virulence-correlated gene	*vcgCPI*	46	40 (87.00)	74.83–94.54	6.67	73.91	0.45	1.000
Virulence-correlated gene	*vcgEP2*	46	0 (0.00)	0–6.3	0.00	− 100	0.00	0.000
Extracellular haemolysin gene	*vfh*	30	21 (70.00)	15.73–47.97	2.33	40	0.51	0.917
Heme utilization protein gene	*hupO*	30	5 (16.70)	6.37–33.15	0.20	− 66	0.04	0.000
Haemolysin toxin	*vfpA*	30	0 (0.00)	0–9.50	0.00	− 100	0.00	0.000
Thermostable direct hemolysin-related gene	*trd*	21	0 (0.00)	0–13.29	0.00	− 100	0.00	0.000
Thermostable direct hemolysin	*trh*	21	0 (0.00)	0–13.29	0.00	− 100	0.00	0.000
Flagellar genes	*flaE*	21	0 (0.00)	0–13.29	0.00	− 100	0.00	0.000
Outer membrane proteins	*ompU*	5	0 (0.00)	0–45.07	0.00	− 100	0.00	0.001
Zonula occludens toxin	*zot*	5	0 (0.00)	0–45.07	0.00	− 100	0.00	0.001
Protease gene	*hylA*	5	0 (0.00)	0–45.07	0.00	− 100	0.00	0.001
Total		316	112 (35.44)					

*CI* confidence interval, *LL* lower limit, *UL* upper limit, *RR* relative risk, *aR* attributed risk.

**Figure 6:**
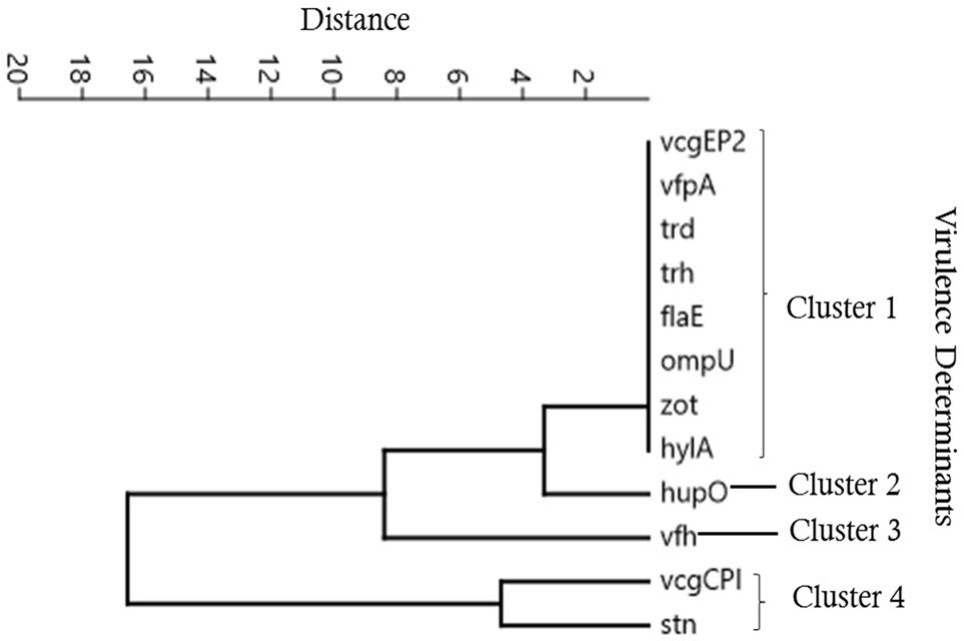
Identified *Vibrio* spp. Virulence determinants contingent on their presence in Surface Waters.

Using the multi-way cluster analysis, the pathogenic species virulence toxins was grouped according to their frequency of occurrence. Four clusters of virulence determinants were distinguished on the base of their frequency of occurrence (percentage number) and the surface water they were identified in. The calculations showed that the virulence toxins (*vcgEP2*, *vfpA*, *trd*, *trh*, *flaE*, *ompU*, *zot*, *hylA*) clustered into no. 1, (0.00%, n = 112); *hupO* clustered into 2, (4.46% n = 112); vfh clustered into no. 3, (18.75%, n = 112); and *vcgCPI* and *stn* clustered into no. 4, (35.71%, and 41.07%, n = 112) Fig. 6. But when converting results by only considering the number of specific virulence toxin contaminations in isolates, the frequence of ocurrence was toxins *vcgEP2*, (0.00% n = 46), *vfpA*, (0.00% n = 30); *trd*, *trh*, *flaE* (0 0.00%, n = 21); *ompU*, *zot*, *hylA* (0.00% n = 5); hupO, (16.70% n = 5); vfh (70.00%, n = 21); vcgCPI and stn (82.10%, 87.00%, n = 46, 40) repectively. The Spearman's coefficient (rho) indicated that statistically significant risk was posed by the virulence toxins stn (rho = 0.001) and/or *vcgCPI* (rho = 0.007), *vfh* (rho = 0.037) in surface water sources while for *hupO* (rho = 0.119) and in the case of other toxins the Spearman's coefficient were statistically insignificant.

### Correlations patterns between *Vibrio* spp. virulence toxins and surface water sources

**Figure 7:**
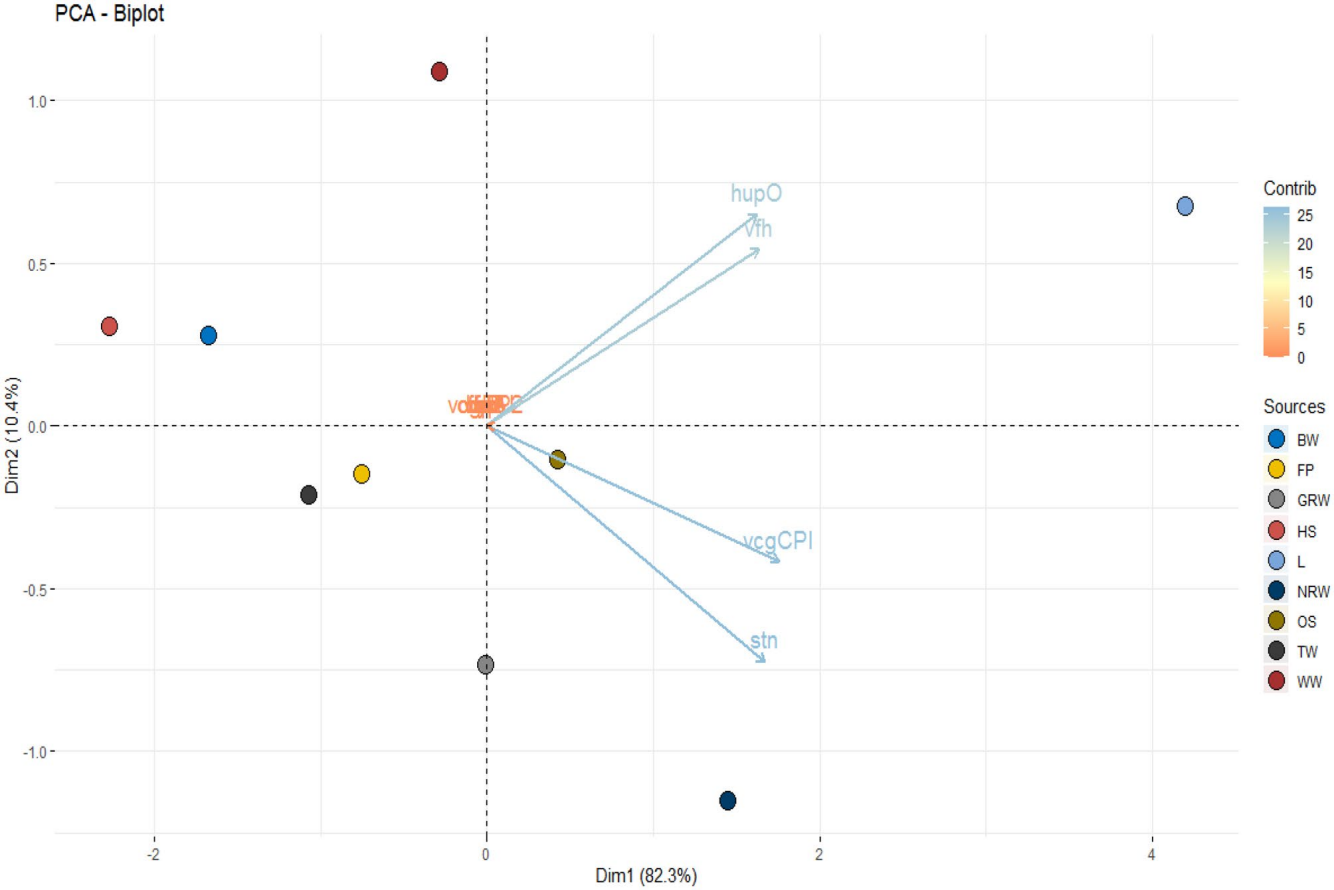
PCA biplot for correlations between the characterised *Vibrio* spp. Virulence determinants and Surface Waters. *BW* bohole water, *FP* fish pond, *GRW* ground running water, *HS* hot spring, *L* Lake, *NRW* natural raw water, *OS* open springs, *TW* tap water, *WW* well water. A PCA biplot for correlating the water sources and the virulence toxins of *Vibrio* spp., the various colour shows their prevalence and dispersion.

We use principal component analysis PCA to study the multivariate association between the distribution of Virulence toxins and Surface waters sources. The results obtained from the PCA in Fig. 7 showed correlations between virulence toxins level contamination and Surface water sources of the isolated *Vibrio* spp. Interestingly, the Vibriosis virulence toxins (*trd*, *vfpA*, *trh*, *vcgEP*, *zot*, *flaE*, *ompU*) show no correlation with the isolates recovered from the water sources. On the other hand, the toxins (*hupO*, *vfh*) are positively correlated with the isolate recovered from the bohole water (BW) source. Similarly, (*vcgCPI*, *stn*) toxins are positively correlated with natural raw water (NRW) and Open Springs (OS) water sources isolates.

To better understand the result, details of variables contained in PCA1 and PCA2, which explain the total correlation of 91.7%, were analysed further as recommended by scree plot of eigenvalues evaluation (p < 0.05). The PCA1 accounts for 82.3% variability of which *vcgCPI* is the most strongly corrected, with sufficient correlation significant (r = 0.9531264 and p. value of 0.00007), *stn* (r = 0.9059934 and p. value of 0.00076), *vfh* (r = 0.8876029 and p. value of 0.00140) and hupO (r = 0.8807374 and p. value of 0.00171) associated with surface waters isolates. While the PCA2 account for only 10.4%, with insufficient significant correlation in Fig. 7.

## Discussion

For the past decade, an array of several virulence determinants implicated in the pathogenesis of vibriosis have been reported both from clinical and environmental strains across the world. This study screened the prevalence and epidemiological significance of the virulence toxins *stn, vcgCPI, vcgEP2, vfh, hupO, vfpA, trd, trh, flaE, ompU,* and *zot* by PCR. The density of the *Vibrio* bacteria enumerated varies between months and water sources, with the highest densities record in September compared to other months (Fig. 2A–D). This may be due to changes in moisture and poor hygienic practice in some districts where poor probability orientation—also, the majority of the population living in the hard-to-reach region practice open defecation^58^. The geographical location of the sampling sites has a significant contribution to the changing pattern of *Vibrio* densities observed in the respective months. However, the *Vibrio* densities obtained are sufficient to initiate an infection in humans, which is in harmony with the studies of various investigators^54^. In addition, *Vibrio* species quantity (of about 2 × 10^5^ to 3 × 10^7^ CFU/ml) with a cultivation time spanning 4–96 h (approximately 15 h) is sufficient to cause acute gastroenteritis^59,60^ as previously reported by various investigators. Table 4 and Fig. 3 show that the pattern or profile of *Vibrio* strains recovered from the environmental samples depicts the magnitude of pathogenicity seen among *Vibrio* strains. Interestingly, the two major classes of *Vibrio* spp. and its associated infections were adequately represented among the isolates recovered. Thus, cholerae strain (*ompW*) and non-cholera strains (*V. vulnificus* (*toxR*), *V. fluvialis* (*toxR*), *V. parahaemolyticus* (*toxR*), *V. alginolyticus* (*Vg gyrB*), *V. mimicus* (*VM*) were the observed prevalence strains.

Such *Vibrio* spp., infections could be worse in the immune-compromised individual^61^; all the same, the infections are often self-limited^62^. This is also similar to^63^ reports that the worst infection is observed in immune-suppressed patients. Major enterotoxins are expressed by *Vibrio* strains, including *V. cholerae*, *V. paraheamolyticus*, and *V. mimicus*. Interestingly, our result shows a very much high prevalence of 46/56 (82.10%) in the studied water sources as depicted in Table 4 (Figs. 4, 5), compared to the study of^64^, which shows a relatively low frequency of 28.2% amongst non-O1/non-O139 strains. Their study revealed 10.5% of toxigenic *V. cholerae* O1 and 14.3% among O139 serogroups belonging to *Vibrio cholerae*, which are recovered from environmental samples in Europe. In Thailand, 10/21 of clinical isolates were recovered^65^, and 26/193 (13.5%) in *V. fluvialis* of an environmental specimen of South Africa^11^.

All the *Vibrio* strains studied show 35.44% positive to the virulence genes, and specifically, its result is as follows: Lake (27.68%), natural raw water sources (18.75%) and open Springs (13.39%), as shown in Table 5, indicating a zero-tolerance limit for vibriosis infections.

Results show that the virulence toxins include *stn* (82.10%) n = 56, *vcgCPI* (87%) n = 46, *vfh* (70%) n = 21, where the most highly prevalent. Whereas *vcgEP2*, *hupO*, *vfpA*, *trd*, *trh*, *flaE*, *ompU*, *zot*, *hylA* were not detected in the surface waters in the region Fig. 6. Surprisingly, the results show a higher prevalence of *Vibrio* spp., virulence toxins or genes than the report in South Africa—1.0–13.5%^11^, in Europe, *stn*/*sto* genes 28.2%^64^, in Bangladesh *tcpI*, *tcpA*, *ctxA*, and zot (0.2–2%), *hlyA*, *rtxA*, *hap*, and T6SS (82–99%) and T3SS (7–13%) genes^66^. The results indicate the higher risk and potential public health threat of surface water contamination by *Vibrio* spp., virulence toxins. On the other hand, our result was opposite to the findings^67^, where all the *Vibrio* strains were positive for genes *vfh*, *hupO* and *vfpA* negative for gene *stn* encoding the toxin NAG-ST enterotoxin^17^.

The absence of thermostable direct haemolysin (*tdh*) and the *tdh* related haemolysin (*trh*) virulence toxins responsible for the pathogenicity of *V. parahaemolyticus* is in agreement with the recent report of genes for cholerae toxin (*ctx*), thermostable direct hemolysin (*tdh*), or zonula occludens toxin (*zot*) as there were not detected in any of 116 isolates of seawater in Norway^31^. Similar to the study of^68^ Lake isolates in Ohio US, and^69^ *ctxA*, *tcpA*, and *zot* were not detected in the *V. cholerae* strains, while *hlyA*, *rtxA*, and *rtxC* were positive for water sample isolate in China^69^. However, these toxins have been reported in a relatively low occurrence in environmental samples in Malaysian^70^, in Turkey^9^ in Europe and Atlantic coast in Spain^71^, in Italy^72^. Also, it has been reported that the highly cytotoxic and human gastrointestinal infecting *Vibrio* parahaemolyticus strains of environmental origin with no detection of the tdh or trh genes^10^ were observed. We are not surprised about the difference and absence of some virulence toxins in the *Vibrio* strains. Most virulence toxins predominate in clinical isolate sources of toxigenic vibriosis, e.g. *ctxAB* or *tdh* and *trh* are predominant in a clinical strain of *V. cholera* and *V. parahaemolyticus*^17^. Although Virulence toxins/genes *hupO*, *vfh* and *vfpA* are often detected in *V. fluvialis* of both patient isolates, and seafood isolates strains, some *Vibrio* strains virulence can be prevalent irrespective of the origin.

The *Vibrio* strains may have acquired the virulence toxins *stn* by horizontal gene transfer or natural genetic exchange by Organism interactions in the ecosystems or human host^73,74^. The significant role of *stn* is unclear in vibriosis pathogenesis (Table 5). However, the high frequency of concern is a threat to the population using the water sources.

The result of the virulence-correlated gene (vcg) of *Vulnificus*, *vcgCPI* for clinical (C-) genotypes and *vcgEP2* environmental (E-) genotypes were observed to *vcgEP2* 40/46 (87%), and *vcgCPI* 0/46 (0%) is similar to the findings of^75^ (46.9%) 137 of E genotype and (53.1%) 155 of C genotype Of the 292 isolates recovered from water samples, and also related to the study of^11^ of 6/74 (8.1%) had the *vcgC*, and 68/74 *vcgE* (91.9%).

However, it is surprising to find clinical genotypes highly prevalent in the surface waters in our study while there are no environmental isolates detected. Also, we cannot say if these *V. vulnificus* isolates indicate less or high virulent strains, as it has been reported that the *vcgC* gene linked to clinical isolates is potentially more virulent *vcgE* linked to environmental isolates is less virulent^76^. It also implies a difference to the report where almost an equal per cent of *vcgE* (46.9%) and *vcgC* (53.1%) were detected from oyster isolates^77^ and water areas surrounding oyster harvest^75^. Consequently, it is of necessity to continuously monitor surface water source uses, although *Vulnificus* infection is frequent in aquaculture and rare in humans but can be fatal in immunocompromised persons, causing wound ulceration infections, gastroenteritis or septicemia.

The result also showed a prevalence of 21/30 (70%) of *vfh* genes in the *Vibrio* flavilis strains, depicting health as significant as the virulence phenotypes were predominant in this species. *Vibrio* cholera and *Vibrio vulnificus*^78,79^ utilised 70% of *vfp* toxin acting as homologous precursor proteins of metalloproteases during pathogenicity in humans. Specifically, *Vfh* expressed by *Vibrio vulnificus* protease is implicated in proteolytic activity as well in haemagglutinating enhancing permeability and haemorrhagic activities^17,80^. Also, the report showed that *vfp* virulence is predominant in *Vibrio* strains of clinical origin of and the expression of these toxins could be more virulent in their pathogenesis^17^.

Applying multi-way statistical computations provides a novel technique to interpret data on the prevalence of virulent toxins and contaminations levels associated with the water sources. Food and water research rarely adopt such calculations, except in few studies^57,81,82^. However, this approach has not been untaken in literature; it gave us a more in-depth characteristic of *Vibrio* virulence toxin prevalence in the water samples. The multi-way computations enabled us to identify *stn* and *vcgCPI* as the most frequent toxins occurrence in sources terms of percentages. The PCA results indicate the prevalence of toxins in correlations to their sources; the analysed shown *hupO* and *vfh* are associated with bohole water (BW) source while *vcgCPI*, and *stn*, are positively correlated with natural raw water (NRW) and open springs (OS) water (Fig. 7). This study has expanded the baseline databases for *Vibrio* spp., and associated infections in this region. Therefore, the findings of this study have provided the basis for future studies lasting up to 3 years design to establish a trend.

## Conclusion

In this study, the analysed *Vibrio* spp., recovered from the water sources used in the region of Uganda, were found to harbour virulence toxins of significant potential health concern. This is concerned with causing diseases associated explicitly with diarrhoeagenic infections, septicemia, and outbreaks of vibriosis in the region where there is an inadequate water supply or water treatment. The heat-stable enterotoxin (*stn*) and *V. vulnificus* virulence genes (*vcgCPI*) were the most frequently occurring toxins in Lakes and natural raw water in the region. The use of computational analysis turned out to be an effective tool in evaluating the distribution of *Vibrio* spp., virulence toxin in water used in the studied districts. The first study specifically evaluated the prevalence of *V. vulnificus,* *V. fluvialis,* *V. parahaemolyticus,* *V. alginolyticus,* and *V. mimicus* among *Vibrio* spp., and the associated virulence toxins from water sources in South West of Uganda to the best of our knowledge. Interestingly, the findings highlight the pathogenicity and epidemiological characteristic of virulence toxins to enhance surveillance data and its epidemic-causing potential. It provided scientific evidence for the prevalence and distribution of virulence toxins in water, which is of great importance to preventing vibriosis infections and outbreaks. Improved personal and environmental hygiene, including water sanitation practices, is highly recommended.

## Supplementary Information

Supplementary Information.

## References

[1] Osunla CA, Okoh AI (2017). Vibrio pathogens: A public health concern in rural water resources in sub-Saharan Africa. Int. J. Environ. Res. Public Health.

[2] Nezhad SY, Jazani RK, Jahangiri K (2019). Effective factors on outbreaks of food and water borne diseases in Iran: A trend analysis. Indian J. Forensic Med. Toxicol..

[3] Igbinosa EO, Okoh AI (2008). Emerging Vibrio species: An unending threat to public health in developing countries. Res. Microbiol..

[4] Eyisi OAL, Nwodo UU, Iroegbu CU (2013). Distribution of Vibrio species in shellfish and water samples collected from the Atlantic coastline of South-East Nigeria. J. Heal. Popul. Nutr..

[5] Goforth JB, Walter NE, Karatan E (2013). Effects of polyamines on *Vibrio cholerae* virulence properties. PLoS ONE.

[6] Kitamoto S, Nagao-Kitamoto H, Kuffa P, Kamada N (2016). Regulation of virulence: The rise and fall of gastrointestinal pathogens. J. Gastroenterol..

[7] Renee Bina X, Taylor DL, Vikram A, Ante VM, Bina JE (2013). *Vibrio cholerae* ToxR downregulates virulence factor production in response to cyclo(Phe-Pro). MBio.

[8] Terzi G, Büyüktanir Ö, Yurdusev N (2009). Detection of the tdh and trh genes in Vibrio parahaemolyticus isolates in fish and mussels from Middle Black Sea Coast of Turkey. Lett. Appl. Microbiol..

[9] Terzi Gulel G, Martinez-Urtaza J (2016). Molecular characterizations of *Vibrio parahaemolyticus* in seafood from the Black Sea, Turkey. Lett. Appl. Microbiol..

[10] Raghunath P (2014). Roles of thermostable direct hemolysin (TDH) and TDH-related hemolysin (TRH) in *Vibrio parahaemolyticus*. Front. Microbiol..

[11] Fri J, Ndip RN, Njom HA, Clarke AM (2017). Occurrence of virulence genes associated with human pathogenic Vibrios isolated from two commercial Dusky Kob (*Argyrosmus japonicus*) farms and Kareiga estuary in the Eastern Cape Province, South Africa. Int. J. Environ. Res. Public Health.

[12] Sangeetha MS, Shekar M, Venugopal MN (2017). Occurrence of clinical genotype *Vibrio vulnificus* in clam samples in Mangalore, Southwest coast of India. J. Food Sci. Technol..

[13] Todar. *Vibrio cholerae*. *2005* http://textbookofbacteriology.net/cholera_2.html (2005).

[14] Jones MK, Oliver JD (2009). *Vibrio vulnificus*: Disease and pathogenesis. Infect. Immun..

[15] Chakraborty R (2006). Species-specific identification of *Vibrio fluvialis* by PCR targeted to the conserved transcriptional activation and variable membrane tether regions of the toxR gene [3]. J. Med. Microbiol..

[16] Tall BD (2003). Characterization of *Vibrio fluvialis*-like strains implicated in limp lobster disease. Appl. Environ. Microbiol..

[17] Liang P, Cui X, Du X, Kan B, Liang W (2013). The virulence phenotypes and molecular epidemiological characteristics of *Vibrio fluvialis* in China. Gut Pathog..

[18] Ramamurthy T, Chowdhury G, Pazhani GP, Shinoda S (2014). *Vibrio fluvialis*: An emerging human pathogen. Front. Microbiol..

[19] Huang KC, Hsu RWW (2005). *Vibrio fluvialis* hemorrhagic cellulitis and cerebritis. Clin. Infect. Dis..

[20] Ratnaraja N, Blackmore T, Byrne J, Shi S (2005). *Vibrio fluvialis* peritonitis in a patient receiving continuous ambulatory peritoneal dialysis. J. Clin. Microbiol..

[21] Rodríguez, L. E. C. *et al.* Severe otitis due to *Vibrio fluvialis* in a patient with AIDS: First report in the world. *Rev. Cubana Med. Trop.* (2005). 17966587

[22] Liu WL, Chiu YH, Chao CM, Hou CC, Lai CC (2011). Biliary tract infection caused by *Vibrio fluvialis* in an immunocompromised patient. Infection.

[23] Lai CH (2006). Severe watery diarrhoea and bacteraemia caused by *Vibrio fluvialis*. J. Infect..

[24] Penland RL, Boniuk M, Wilhelmus KR (2000). Vibrio ocular infections on the US Gulf Coast. Cornea.

[25] Noriega-Orozco, L., Acedo-Félix, E., Higuera-Ciapara, I., Jiménez-Flores, R. & Cano, R. Pathogenic and non pathogenic Vibrio species in aquaculture shrimp ponds. *Rev. Latinoam. Microbiol.* (2008).

[26] Vandenberghe J, Thompson FL, Gomez-Gil B, Swings J (2003). Phenotypic diversity amongst Vibrio isolates from marine aquaculture systems. Aquaculture.

[27] Matsuda S, Okada N, Kodama T, Honda T, Iida T (2012). A cytotoxic type III secretion effector of vibrio parahaemolyticus targets vacuolar H+-ATPase subunit C and ruptures host cell lysosomes. PLoS Pathog..

[28] Wang, R. *et al.* The pathogenesis, detection, and prevention of *Vibrio parahaemolyticus*. *Front. Microbiol*. **6** (2015). 10.3389/fmicb.2015.00144 PMC4350439 25798132

[29] Bhattacharjee, S. *et al.* Is *Vibrio fluvialis* emerging as a pathogen with epidemic potential in coastal region of eastern India following cyclone Aila? *J. Heal. Popul. Nutr.* (2010). 10.3329/jhpn.v28i4.6036 PMC2965321 20824973

[30] Bharati, K. & Ganguly, N. K. Cholera toxin: A paradigm of a multifunctional protein. *Indian J. Med. Res*. (2011). PMC3089049 21415492

[31] Håkonsholm F (2020). Vibrios from the Norwegian marine environment: Characterization of associated antibiotic resistance and virulence genes. Microbiologyopen.

[32] Kehlet-Delgado H, Häse CC, Mueller RS (2020). Comparative genomic analysis of Vibrios yields insights into genes associated with virulence towards *C. gigas* larvae. BMC Genom..

[33] Vezzulli L (2016). Climate influence on Vibrio and associated human diseases during the past half-century in the coastal North Atlantic. Proc. Natl. Acad. Sci. USA..

[34] Bwire G (2017). Identifying cholera ‘hotspots’ in Uganda: An analysis of cholera surveillance data from 2011 to 2016. PLoS Negl. Trop. Dis..

[35] MOH-Uganda. Annual Health Sector Performance Report FY 2017/2018. *Repub. Uganda* (2019).

[36] WHO (2013). WHO|Guidelines for Drinking-Water Quality.

[37] UNICEF, IFRC & WHO (2020). Focus Group Discussion Guide for Communities.

[38] QGIS Development Team. QGIS Geographic Information System. Open Source Geospatial Foundation Project. *Qgisorg*. http://qgis.osgeo.org. (2014).

[39] Adefisoye MA, Okoh AI (2016). Identification and antimicrobial resistance prevalence of pathogenic *Escherichia coli* strains from treated wastewater effluents in Eastern Cape, South Africa. Microbiologyopen.

[40] Pfeffer C, Oliver JD (2003). A comparison of thiosulphate-citrate-bile salts-sucrose (TCBS) agar and thiosulphate-chloride-iodide (TCI) agar for the isolation of Vibrio species from estuarine environments. Lett. Appl. Microbiol..

[41] Kriem MR (2015). Prevalence of *Vibrio* spp. in raw shrimps (*Parapenaeus longirostris*) and performance of a chromogenic medium for the isolation of Vibrio strains. Lett. Appl. Microbiol..

[42] Igere BE, Okoh AI, Nwodo UU (2020). Antibiotic susceptibility testing (AST) reports: A basis for environmental/epidemiological surveillance and infection control amongst environmental vibrio cholerae. Int. J. Environ. Res. Public Health.

[43] Maugeri TL, Carbone M, Fera MT, Gugliandolo C (2006). Detection and differentiation of *Vibrio vulnificus* in seawater and plankton of a coastal zone of the Mediterranean Sea. Res. Microbiol..

[44] Kwok AYC (2002). Phylogenetic study and identification of human pathogenic Vibrio species based on partial hsp60 gone sequences. Can. J. Microbiol..

[45] Haldar S (2010). Development of a haemolysin gene-based multiplex PCR for simultaneous detection of *Vibrio campbellii*, *Vibrio harveyi* and *Vibrio parahaemolyticus*. Lett. Appl. Microbiol..

[46] Goel AK, Ponmariappan S, Kamboj DV, Singh L (2007). Single multiplex polymerase chain reaction for environmental surveillance of toxigenic—pathogenic O1 and non-O1 *Vibrio cholerae*. Folia Microbiol. (Praha.).

[47] Chakraborty R (2005). Cytotoxic and cell vacuolating activity of *Vibrio fluvialis* isolated from paediatric patients with diarrhoea. J. Med. Microbiol..

[48] Zhou S, Hou Z, Li N, Qin Q (2007). Development of a SYBR Green I real-time PCR for quantitative detection of *Vibrio alginolyticus* in seawater and seafood. J. Appl. Microbiol..

[49] Adefisoye MA, Okoh AI (2017). Ecological and public health implications of the discharge of multidrug-resistant bacteria and physicochemical contaminants from treated wastewater effluents in the Eastern Cape, South Africa. Water (Switzerland).

[50] Abdelaziz M, Ibrahem MD, Ibrahim MA, Abu-Elala NM, Abdel-moneam DA (2017). Monitoring of different vibrio species affecting marine fishes in Lake Qarun and Gulf of Suez: Phenotypic and molecular characterization. Egypt. J. Aquat. Res..

[51] Beshiru A, Okareh OT, Okoh AI, Igbinosa EO (2020). Detection of antibiotic resistance and virulence genes of Vibrio strains isolated from ready-to-eat shrimps in Delta and Edo States, Nigeria. J. Appl. Microbiol..

[52] Igere BE, Okoh AI, Nwodo UU (2020). Wastewater treatment plants and release: The vase of Odin for emerging bacterial contaminants, resistance and determinant of environmental wellness. Emerg. Contaminants.

[53] Kim YB (1999). Identification of *Vibrio parahaemolyticus* strains at the species level by PCR targeted to the toxR gene. J. Clin. Microbiol..

[54] Rosche TM, Yano Y, Oliver JD (2005). A rapid and simple PCR analysis indicates there are two subgroups of *Vibrio vulnificus* which correlate with clinical or environmental isolation. Microbiol. Immunol..

[55] Rstudio Team (2019). RStudio: Integrated Development for R.

[56] Abramson JH (2011). WINPEPI updated: Computer programs for epidemiologists, and their teaching potential. Epidemiol. Perspect. Innovat..

[57] ONOHUEAN H, Okoh AI, Nwodo UU (2021). Antibiogram signatures of *Vibrio* species recovered from surface waters in South Western districts of Uganda: Implications for environmental pollution and infection control. Sci Total Environ.

[58] Gibson J, Eales K, Nsubuga-Mugga C (2018). Reviewing sanitation in uganda to reach sustainable development goals. Review. Sanitation Uganda Reach Sustain. Dev. Goals.

[59] Ottaviani D (2012). Nontoxigenic *Vibrio parahaemolyticus* strains causing acute gastroenteritis. J. Clin. Microbiol..

[60] Theron J, Cilliers J, Du Preez M, Brözel VS, Venter SN (2000). Detection of toxigenic *Vibrio cholerae* from environmental water samples by an enrichment broth cultivation—Pit-stop semi-nested PCR procedure. J. Appl. Microbiol..

[61] Qadri F (2003). Adaptive and inflammatory immune responses in patients infected with strains of *Vibrio parahaemolyticus*. J. Infect. Dis..

[62] Baker-Austin C (2018). *Vibrio* spp. infections. Nat. Rev. Dis. Prim..

[63] Nordin N, Yusof NA, Abdullah J, Radu S, Hushiarian R (2017). A simple, portable, electrochemical biosensor to screen shellfish for *Vibrio parahaemolyticus*. AMB Express.

[64] Rivera ING, Chun J, Huq A, Sack RB, Colwell RR (2001). Genotypes associated with virulence in environmental isolates of *Vibrio cholerae*. Appl. Environ. Microbiol..

[65] Tulatorn S, Preeprem S, Vuddhakul V, Mittraparp-arthorn P (2018). Comparison of virulence gene profiles and genomic fingerprints of *Vibrio cholerae* O1 and non-O1/non-O139 isolates from diarrheal patients in southern Thailand. Trop. Med. Health.

[66] Hasan NA (2013). Distribution of virulence genes in clinical and environmental *Vibrio cholerae* strains in Bangladesh. Appl. Environ. Microbiol..

[67] Bidinost, C. *et al.* Virulence factors of non-O1 non-O139 *Vibrio cholerae* isolated in Córdoba, Argentina. *Rev. Argent. Microbiol.* (2004). 15786867

[68] Daboul J (2020). Characterization of *Vibrio cholerae* isolates from freshwater sources in northwest Ohio. PLoS ONE.

[69] Wang Q (2020). The impact of water intrusion on pathogenic vibrio species to inland brackish waters of China. Int. J. Environ. Res. Public Health.

[70] Faja, O. M., Sharad, A. A., Younis, K. M. & Alwan, M. G. Isolation, detection of virulence genes, antibiotic resistance genes , plasmid profile , and molecular typing among *Vibrio parahaemolyticus* isolated in Malaysian seawater from recreational beaches and fish. **12**, 1140–1149 (2019). 10.14202/vetworld.2019.1140-1149 PMC6702555 31528045

[71] Martinez-Urtaza J (2008). Environmental determinants of the occurrence and distribution of *Vibrio parahaemolyticus* in the rias of Galicia, Spain. Appl. Environ. Microbiol..

[72] Di Pinto A, Ciccarese G, De Corato R, Novello L, Terio V (2008). Detection of pathogenic *Vibrio parahaemolyticus* in southern Italian shellfish. Food Control.

[73] Jiang S, Chu W, Fu W (2003). Prevalence of cholera toxin genes (ctxA and zot) among non-O1/O139 *Vibrio cholerae* strains from Newport Bay, California. Appl. Environ. Microbiol..

[74] Rahman MH (2008). Distribution of genes for virulence and ecological fitness among diverse *Vibrio cholerae* population in a cholera endemic area: Tracking the evolution of pathogenic strains. DNA Cell Biol..

[75] Warner E, Oliver JD (2008). Population structures of two genotypes of *Vibrio vulnificus* in oysters (*Crassostrea virginica*) and seawater. Appl. Environ. Microbiol..

[76] Bier N, Diescher S, Strauch E (2015). Multiplex PCR for detection of virulence markers of *Vibrio vulnificus*. Lett. Appl. Microbiol..

[77] Guerrero A, Gómez Gil Rodríguez B, Wong-Chang I, Lizárraga-Partida ML (2015). Genetic characterization of *Vibrio vulnificus* strains isolated from oyster samples in Mexico. Int. J. Environ. Health Res..

[78] Alav I (2021). Structure, assembly, and function of tripartite efflux and type 1 secretion systems in gram-negative bacteria. Chem. Rev..

[79] Hiyoshi H, Kodama T, Iida T, Honda T (2010). Contribution of *Vibrio parahaemolyticus* virulence factors to cytotoxicity, enterotoxicity, and lethality in mice. Infect. Immun..

[80] Miyoshi S (2013). Extracellular proteolytic enzymes produced by human pathogenic vibrio species. Front. Microbiol..

[81] Pax AP (2019). The characterisation of Mozzarella cheese microstructure using high resolution synchrotron transmission and ATR-FTIR microspectroscopy. Food Chem..

[82] Szymczak B, Szymczak M, Trafiałek J (2020). Prevalence of *Listeria* species and *L. monocytogenes* in ready-to-eat foods in the West Pomeranian region of Poland: Correlations between the contamination level, serogroups, ingredients, and producers. Food Microbiol..

[83] Kaysner, C. A. & Angelo DePaola, J. Bacteriological Analytical Manual Chapter 9: Vibrio. *Adm. US Food Drug* (2004).

[84] Farmer, J. J. & Hickman-Brenner, F. W. The Genera Vibrio and Photobacterium. in *The Prokaryotes* 508–563 (Springer New York, 2006). 10.1007/0-387-30746-x_18.

[85] Silva AJ, Pham K, Benitez JA (2003). Haemagglutinin/protease expression and mucin gel penetration in El Tor biotype *Vibrio cholerae*. Microbiology.

[86] Morris JG, Acheson D (2003). Cholera and Other Types of Vibriosis: A Story of Human Pandemics and Oysters on the Half Shell. Clin. Infect. Dis..

