# Supplementary material for: Epidemiologic potentials and correlational analysis of Vibrio species and virulence toxins from water sources in greater Bushenyi districts, Uganda
Source: Sci Rep. 2021 Nov 17;11:22429. doi: 10.1038/s41598-021-01375-3 (PMC8599681; doi:10.1038/s41598-021-01375-3)
Supplement: Supplementary file 1 — Supplementary Information. [file 41598_2021_1375_MOESM1_ESM.docx]

**Epidemiologic potentials and correlational analysis of *Vibrio* species and virulence toxins from water sources in greater Bushenyi districts, Uganda**

ONOHUEAN Hope^1,2,3^ *, OKOH Anthony I. ^1, 2^ and NWODO UU^1,2^

^1^SA-MRC Microbial Water Quality Monitoring Centre, University of Fort Hare, Alice 5700, South Africa

^2^Applied and Environmental Microbiology Research Group (AEMREG), Department of Biochemistry and Microbiology, University of Fort Hare, Private Bag 1314, Alice, 5700 Eastern Cape, South Africa

^3^Biopharmaceutics unit, Pharmacology and Toxicology, School of Pharmacy, Kampala

International University Western Campus, Uganda

*Corresponding author: Onohuean Hope, Email: onohuean@gmail.com; Tel.: +256753802877

**Supplementary full gel pictures**

**
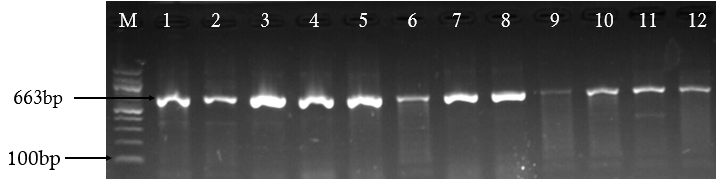
**

Supplementary S1

Gel picture representing molecular confirmation of presumptive isolates using 16S rRNA gene (*Vibrio* genus).

Lane M: ladder (Molecular Marker Thermo-Scientific, 100 bp), lane 1 positive control, lane 2 – 12: Confirmed positive isolates.


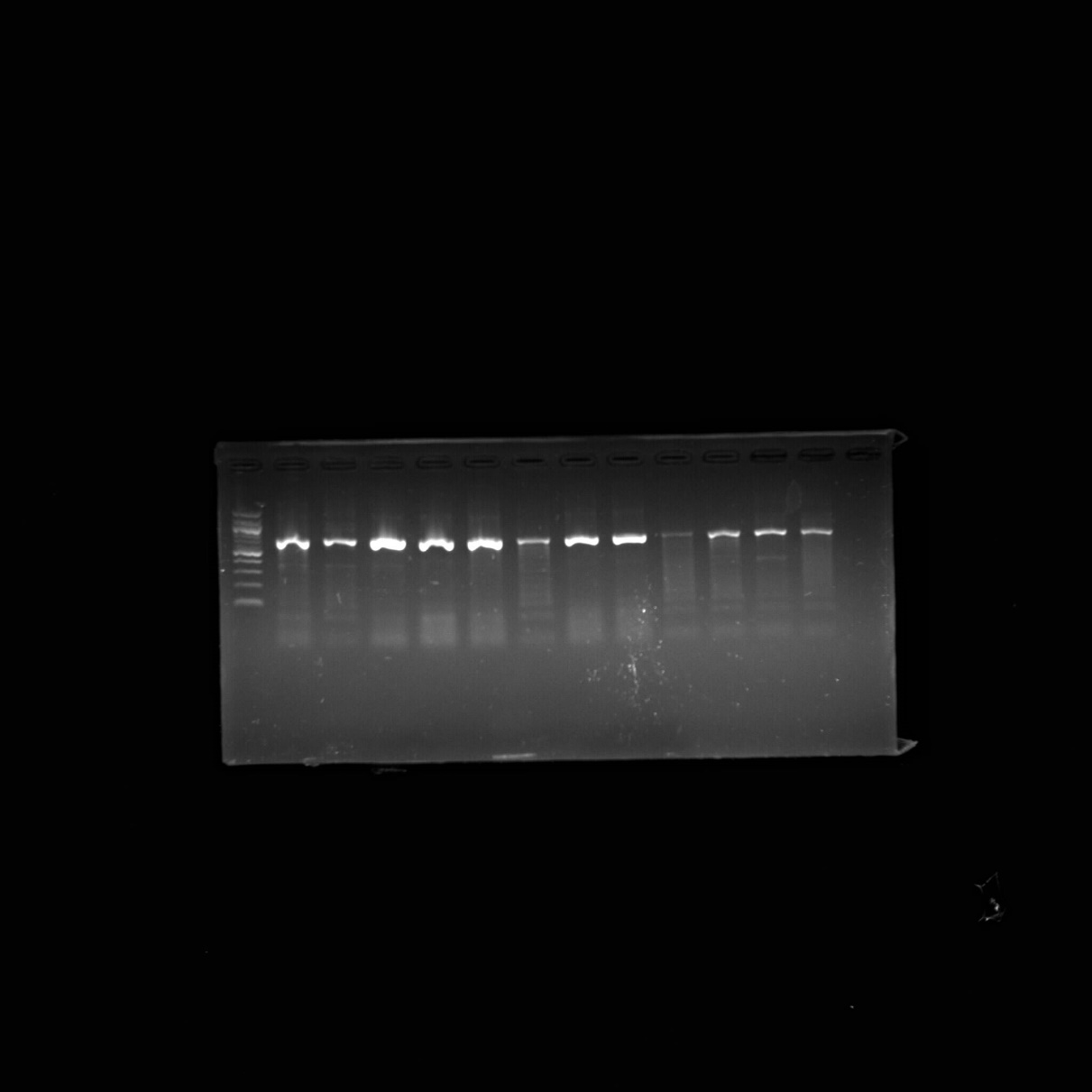


Figure 6

Supplementary S1

Gel picture representing molecular confirmation of presumptive isolates using 16S rRNA gene (Vibrio genus).

**
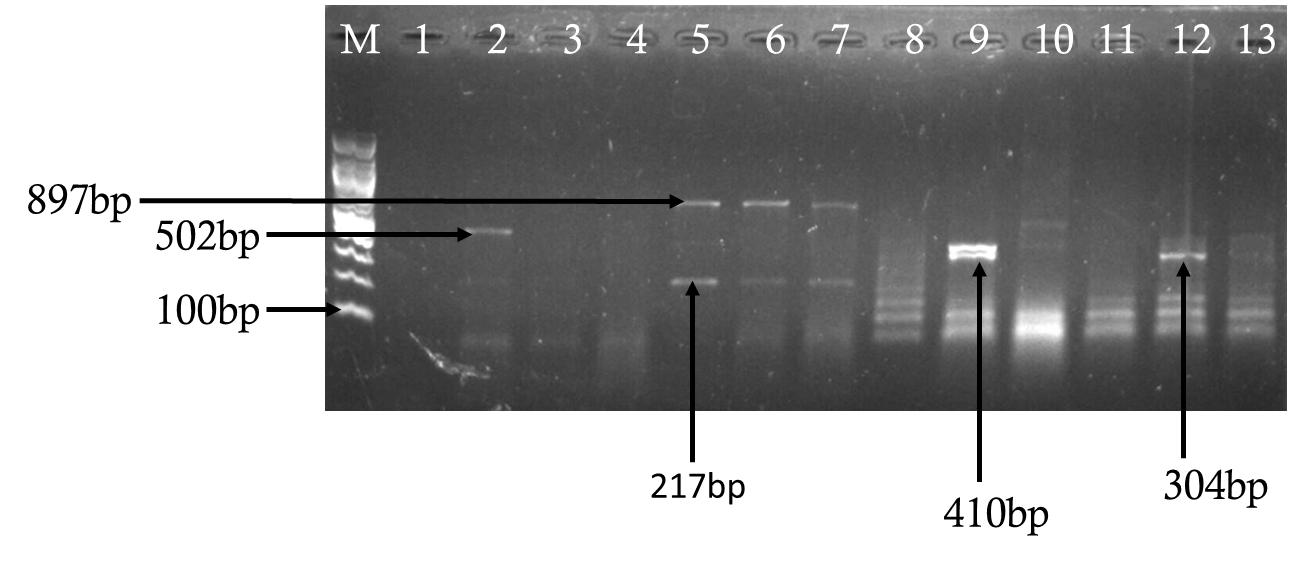
**

Figure 3A


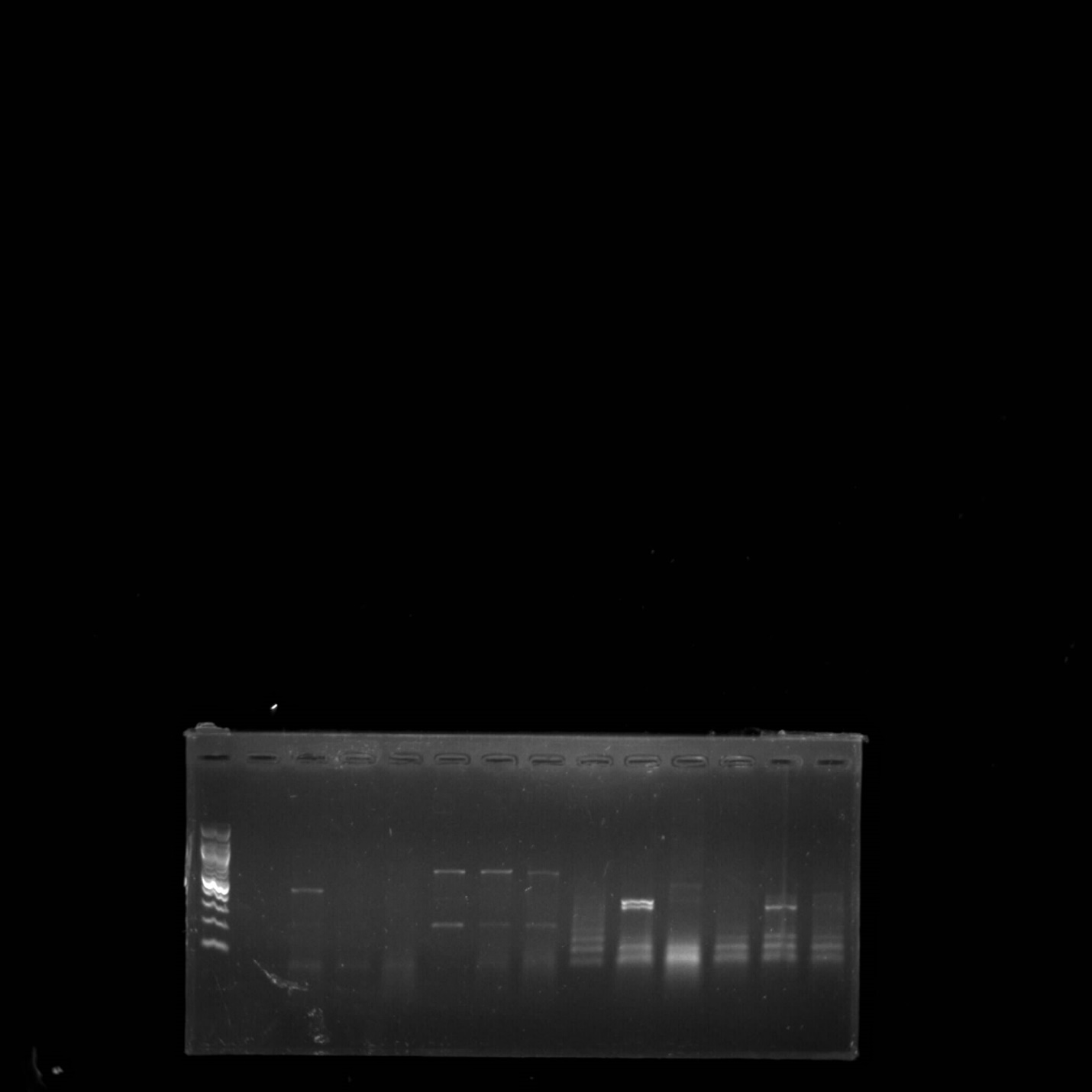


Figure 3A

**
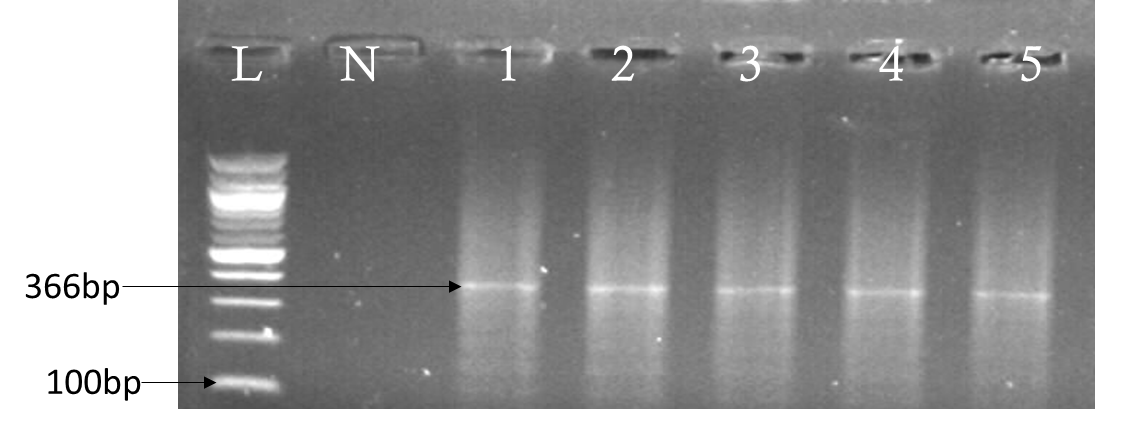
**

Figure 3B


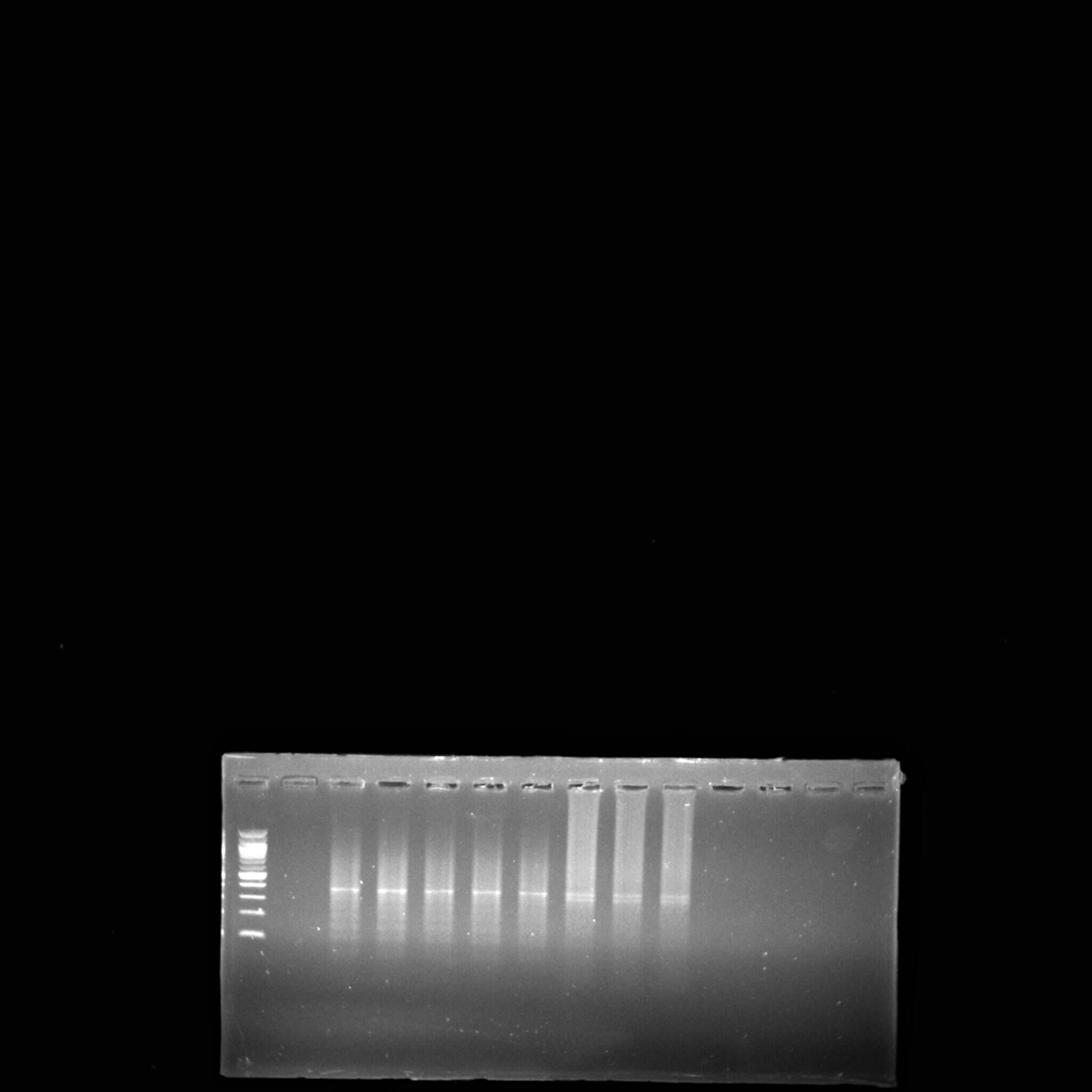


Figure 3B


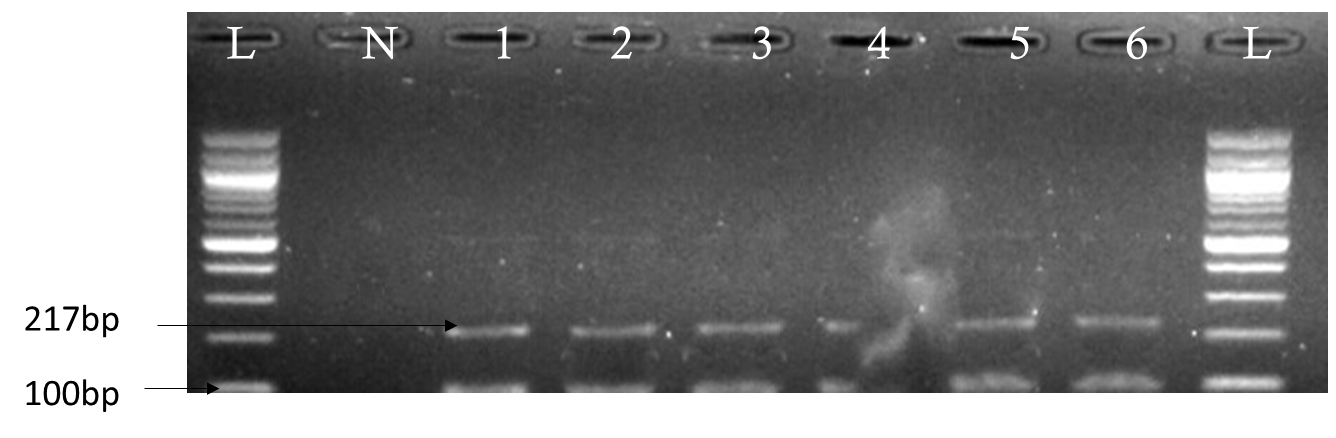


Figure 3C


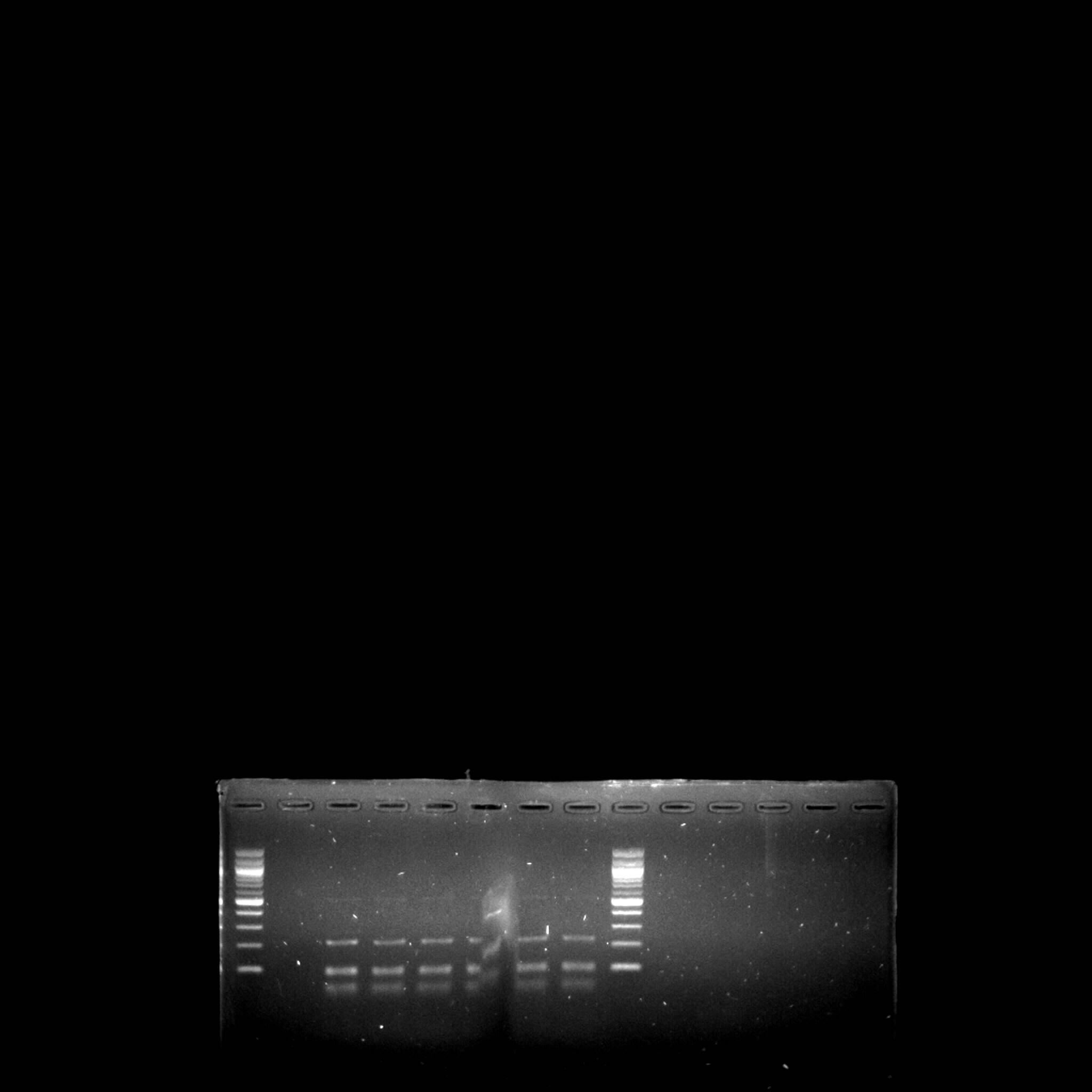


Figure 3C


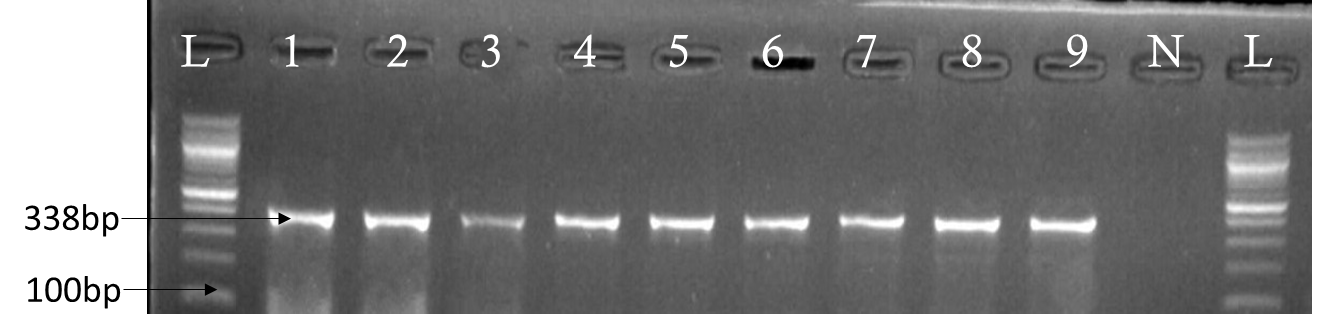


Figure 3D


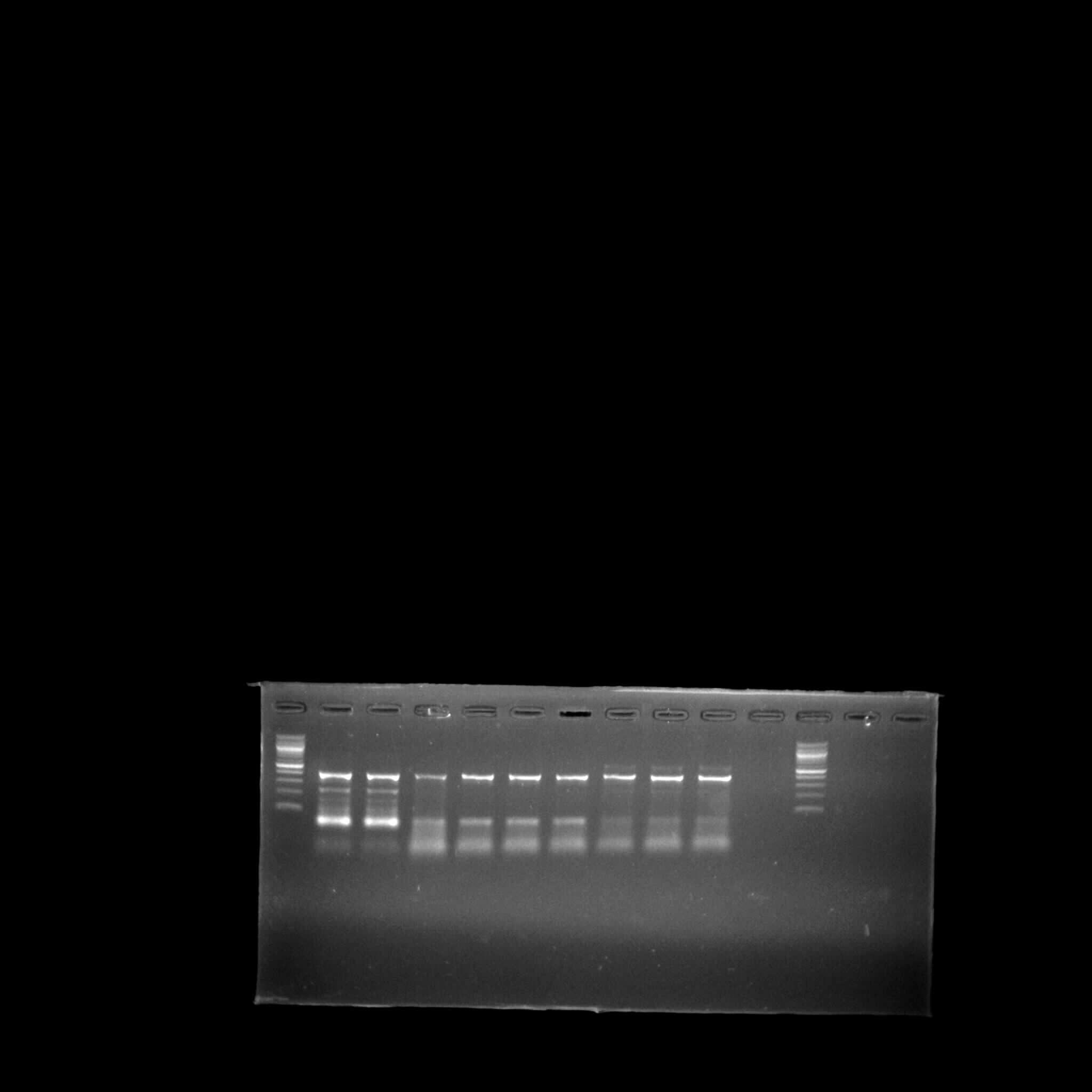


Figure 3D


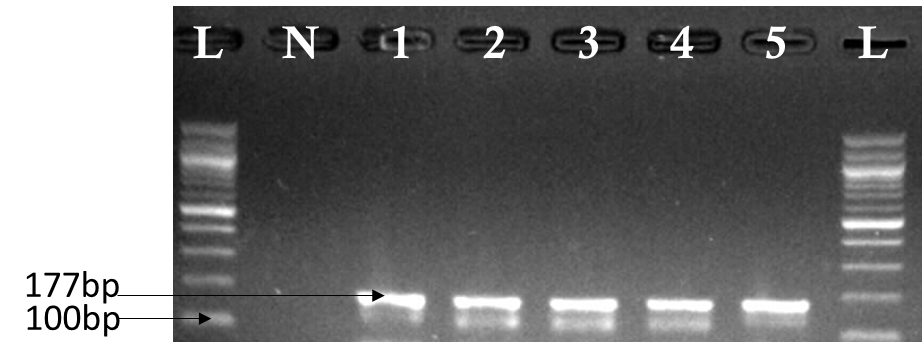


Figure 3E


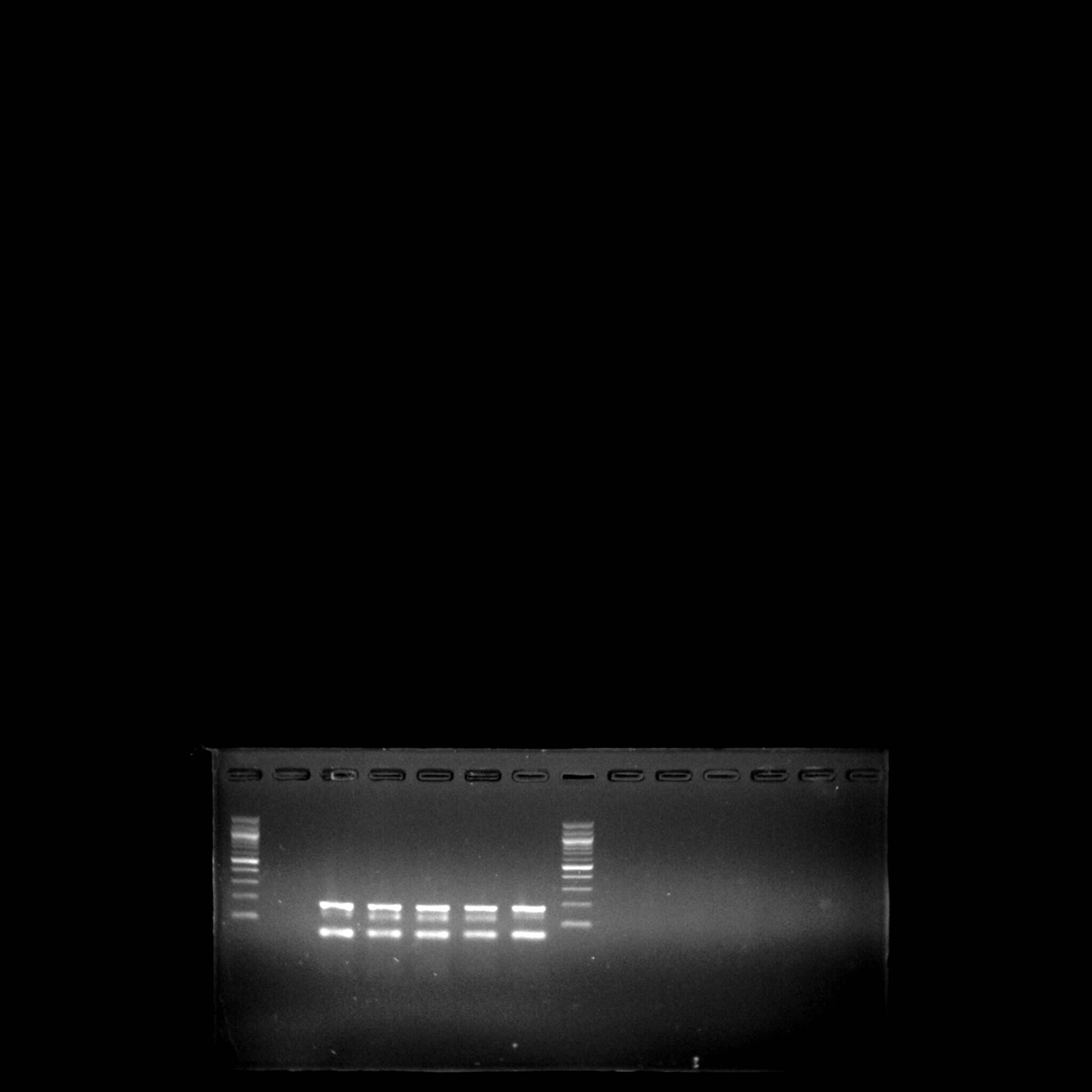


Figure 3E

Figure 3. A-E. Gel picture representing molecular confirmation of the pathogenic Vibrio species.

For A, Lane M: Molecular Marker (100bp); Lane 1: Negative control; Lane 5, 6, 7 (*V. parahaemolyticus (*toxR) and *V. fluvialis* (toxR)); Lane 12, 13: *V. cholerae* (ompW). While B-E, Lane L: Molecular Marker (100bp); Lane N: Negative control and 1 is positive control. B= vulnificus (*vvhA*), C= Fluvialis (*toxR*), D= Alginolyticus (*Vg gyrB*), E=Mimicus (*VM*).

**
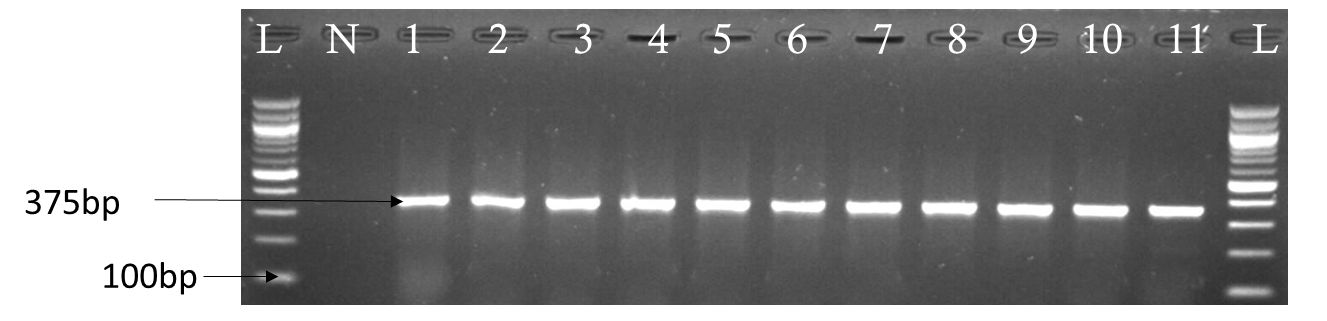
**

Figure 4A


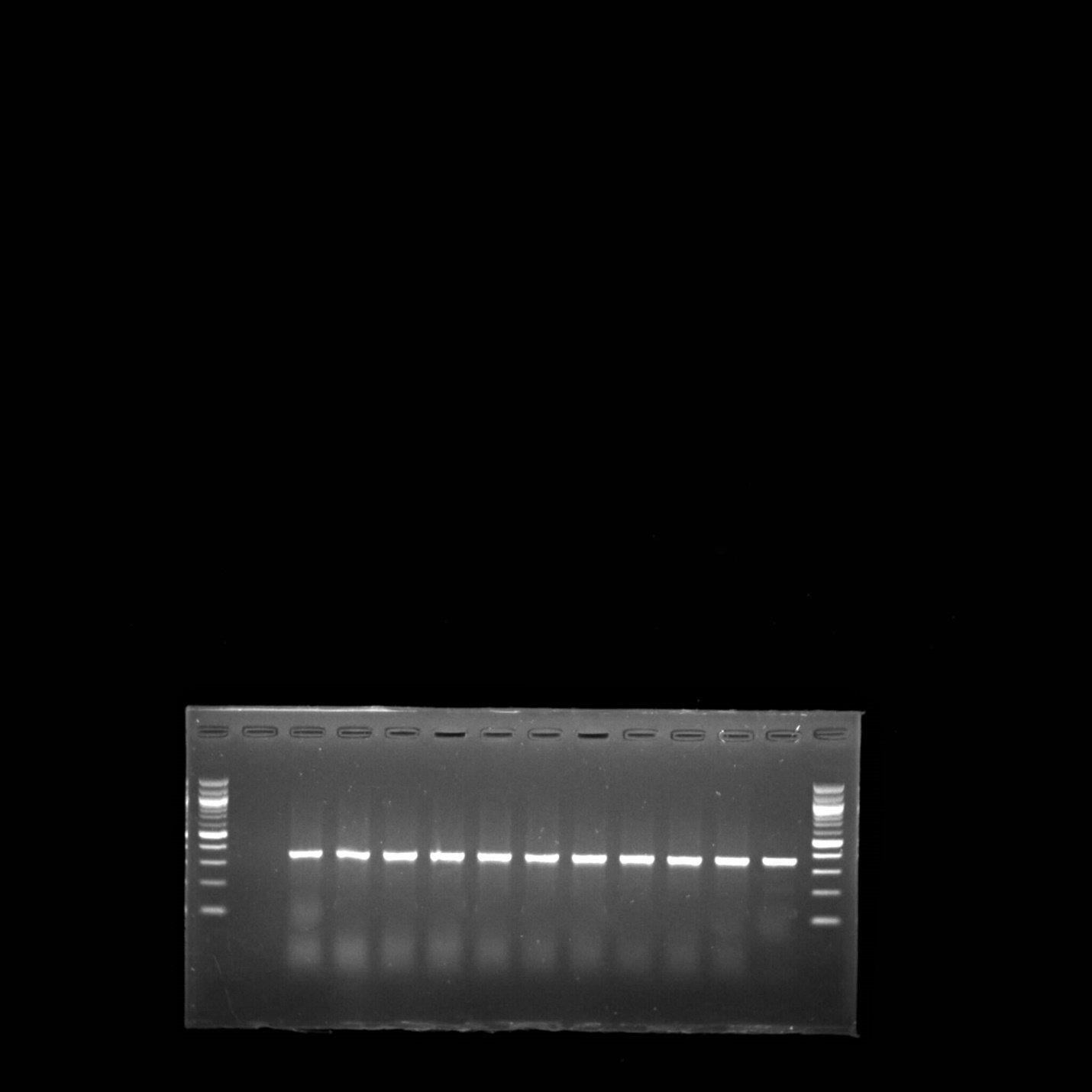


Figure 4A

**
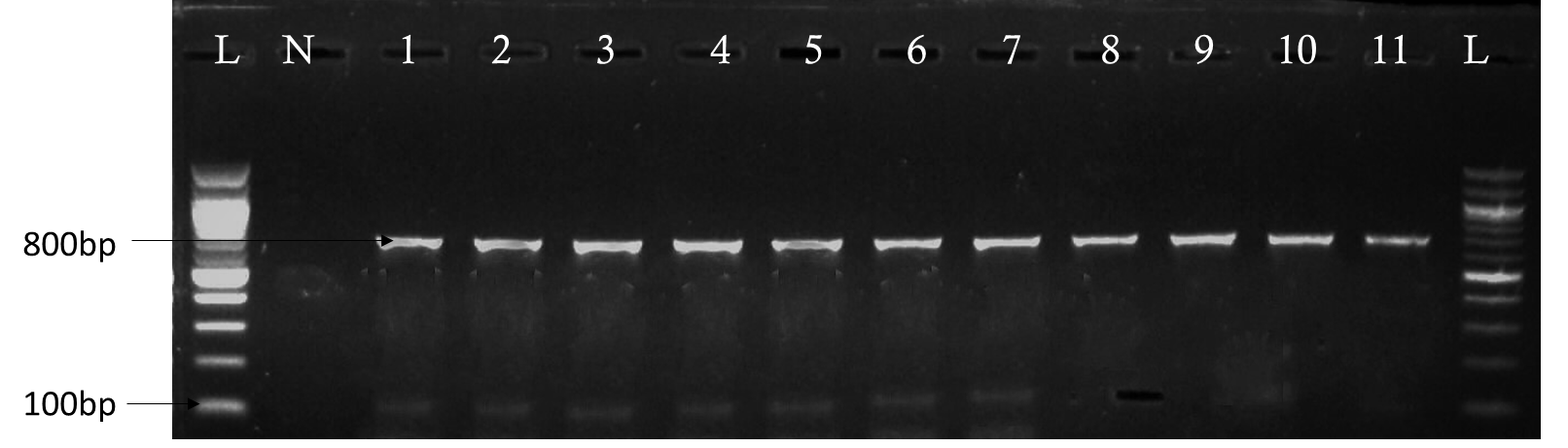
**

Figure 4B


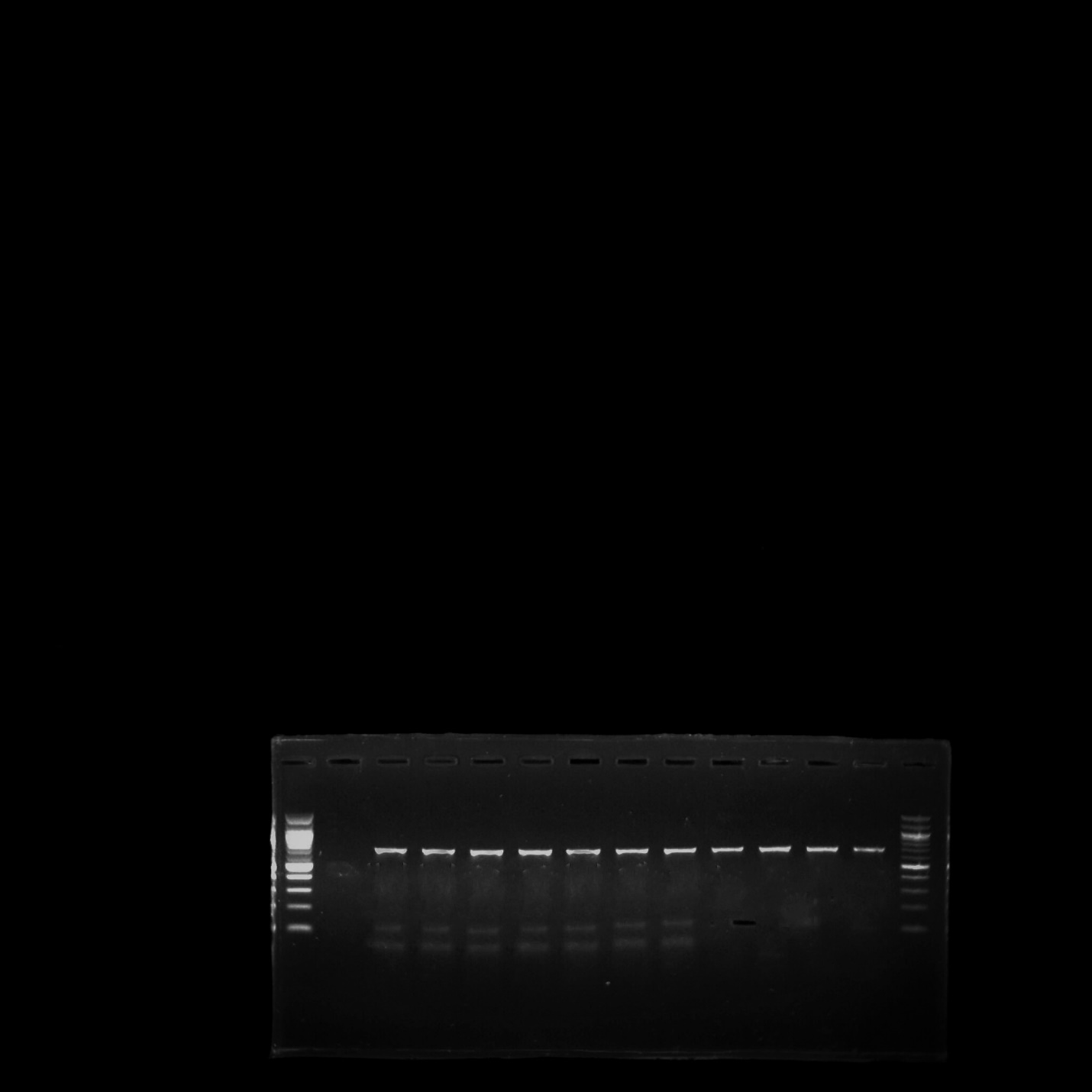


Figure 4B

Figure 4. Gel picture representing molecular characterization of virulence toxins.

A. (*stn* virulence toxins) Lane 1-11 positive, B. (*vfh* virulence toxins) Lane 1-11 positive. Lane L: Molecular Marker (100bp); Lane N: Negative control.
